# Effectiveness and safety of cefiderocol treatment in patients with Gram-negative bacterial infections in Spain in the early access programme: results of the PERSEUS study

**DOI:** 10.1007/s10096-025-05108-6

**Published:** 2025-03-25

**Authors:** Julian Torre-Cisneros, Benito Almirante, Carmen De La Fuente Martos, Pedro Rascado, Miguel Salavert Lletí, Miguel Sánchez-García, Alex Soriano, Maria Cruz Soriano-Cuesta, A. Javier Gonzalez Calvo, Andreas Karas, Jessica Sarda, Stefano Verardi, Ricard Ferrer

**Affiliations:** 1 Maimónides Biomedical Research Institute of Córdoba (IMIBIC), Reina Sofia University Hospital of Córdoba, Córdoba, Spain; 2 Centro de Investigación Biomédica en Red de Enfermedades Infecciosas, Instituto de Salud Carlos III, Madrid, Spain; 3 Infectious Diseases Service, Reina Sofia University Hospital of Córdoba, Córdoba, Spain; 4 Department of Medical and Surgical Sciences, University of Córdoba, Córdoba, Spain; 5 Infectious Diseases Department, Hospital Universitario Vall d’ Hebrón, Barcelona, Spain; 6 Critical Care Service, Hospital Universitario Reina Sofía, Córdoba, Spain; 7 Intensive Care Unit, Complejo Hospitalario Universitario Santiago de Compostela, Santiago de Compostela, Spain; 8 Infectious Diseases Unit, Medical Clinic Department, Hospital Universitario y Politécnico La Fe, Valencia, Spain; 9 Critical Care Department, Hospital Clínico San Carlos, Universidad Complutense, Madrid, Spain; 10 Department of Infectious Diseases, University of Barcelona, Hospital Clinic of Barcelona, Barcelona, Spain; 11 IDIBAPS, Institut d’Investigacions Biomèdiques Agustí-Pi Sunyer, Barcelona, Spain; 12 Intensive Care Medicine Department, Hospital Universitario Ramón y Cajal, Madrid, Spain; 13 Shionogi SLU, Calle de Serrano 45, Madrid, 28001 Spain; 14 Shionogi BV, London, UK; 15 Intensive Care Department, SODIR Reseach Group, Vall d’Hebron Institut de Recerca, Hospital Universitari Vall d’Hebrón, Universitat Autònoma de Barcelona, Barcelona, Spain

**Keywords:** Cefiderocol, Carbapenem resistance, Early appropriate therapy, Multidrug resistance, Gram-negative, *Pseudomonas aeruginosa*, Limited treatment options

## Abstract

**Purpose:**

We assessed the effectiveness and safety of cefiderocol in patients with Gram-negative bacterial infections, excluding *Acinetobacter* spp., in the early access programme (EAP) in Spain.

**Methods:**

The retrospective, multicentre PERSEUS study (2018–2022) enrolled hospitalised patients with serious Gram-negative infections, except *Acinetobacter* spp., who received first-time cefiderocol for ≥ 72 h following requests through the EAP. Clinical cure at end of treatment, all-cause mortality at Day 28, cefiderocol use, and adverse drug reactions (ADRs) were the key outcomes.

**Results:**

Overall, 261 patients were eligible for analysis. Median (interquartile range) age was 61 (49–68) years, 202 (77.4%) were male and 165 (63.2%) were in the intensive care unit. The most frequent diagnoses were respiratory tract infection (47.9%), intra-abdominal infection (14.6%), and urinary tract infection (14.6%). The median (IQR) duration of cefiderocol treatment was 10 (7–14) days. Overall, the clinical cure rate was 80.5% (210/261) and the 28-day mortality rate was 21.5% (56/261). In patients with *Pseudomonas aeruginosa* infection (66.7% [*n* = 174], including 73 [42%] with metallo-β-lactamases), the clinical cure rate was 84.5% (147/174) and the 28-day mortality was 17.2% (30/174). Logistic regression analysis showed that prior antibiotic treatment for > 7 days (OR 0.19, 95% CI 0.05–0.56) and mechanical ventilation (OR 0.32, 95% CI 0.15–0.67) were independent negative predictive factors for clinical cure. ADRs occurred in seven patients, six events resolved, and one was fatal (toxic epidermal necrolysis).

**Conclusions:**

Cefiderocol is a valuable option in the treatment of serious Gram-negative bacterial infections, particularly for those caused by *P. aeruginosa*.

**ClinicalTrials.gov:**

NCT05789199 (Registration date: 16 February 2023).

**Supplementary Information:**

The online version contains supplementary material available at 10.1007/s10096-025-05108-6.

## Introduction

Antibiotic resistance in Gram-negative bacteria has become a major threat to public health globally [1, 2]. Patients with carbapenem-resistant Gram-negative bacterial infections frequently receive inappropriate antimicrobial treatment, which may be associated with increased risk of morbidity and mortality [3, 4]. More specifically, mortality among these patients is in part the consequence of suboptimal empirical and delayed targeted antibiotic therapy [4, 5, 6, 7]; however, other factors, including age and severity of illness are also significant factors [5, 6]. Despite the recent approval of β-lactam–β-lactamase inhibitors and cefiderocol, treatment options remain limited for multidrug-resistant (MDR) and difficult-to-treat resistant (DTR) *Pseudomonas aeruginosa*, *Stenotrophomonas maltophilia*, carbapenem-resistant (CR) *Acinetobacter baumannii* or CR Enterobacterales [8, 9, 10, 11].

Cefiderocol, a siderophore cephalosporin, has potent in vitro activity against a range of aerobic Gram-negative bacteria, including the abovementioned non-fermenters and Enterobacterales [12, 13, 14, 15, 16, 17, 18, 19, 20, 21]. Cefiderocol was approved in Europe in 2020 for the treatment of adult patients with infections caused by susceptible Gram-negative pathogens with limited treatment options and not confined to specific infection site(s) [22]. Cefiderocol was available for the treatment of patients with life-threatening infections under compassionate use before April 2020, and after approval through an early access programme (EAP) in European countries prior to its commercialisation in individual countries [23, 24, 25]. Patients with Gram-negative bacterial infections were eligible to access cefiderocol through the EAP when alternative treatment options were not available otherwise due to resistance, according to the local susceptibility and antibiograms, and/or potential adverse events. Physicians frequently requested cefiderocol as a last-resort antibiotic treatment for critically ill patients infected by non-fermenters [24, 25]. Patients with CR *A. baumannii* infections, who were treated with cefiderocol in the EAP, were enrolled into the retrospective ARES study in European countries, including Spain, therefore were excluded from the current study [25].

The main objectives of the PERSEUS study were to describe the use of cefiderocol in hospitalised patients with serious Gram-negative bacterial infections in the EAP in Spain, and to assess the effectiveness of cefiderocol treatment and adverse drug reactions in this patient population. The current manuscript highlights the outcomes with cefiderocol treatment overall and in patients with specific Gram-negative pathogens, such as *P. aeruginosa* infections and other species. As the study enrolled patients only from the EAP, the analyses also aimed to investigate any association between timing of cefiderocol treatment and clinical outcomes.

## Methods

### Ethics and regulations

Access to locally unapproved medications could be granted under special circumstances in Spain (Royal Decree 1015/2009) and approved on a case-by-case basis by the Spanish Agency of Medicines. The study was approved by the institutional review board of Hospital La Princesa, Madrid, on 3 November 2020 (Royal Decree 957/2020), which served as central reference ethics committee. The study was compliant with all legal and regulatory requirements, the International Conference on Harmonisation Good Clinical Practice E6 guidelines and the Declaration of Helsinki.

Only anonymised data were collected from patients’ medical records. According to Spanish regulations, the informed consent for participants was waived by the regulator because patients completed their treatment prior to initiation of this retrospective study and the study represented no harm for the participants.

### Study design

The PERSEUS study was a retrospective, multicentre, observational, medical chart review study in patients with Gram-negative bacterial infections, excluding all *Acinetobacter* spp. infections, through the Shionogi compassionate use programme and EAP in Spain (2018–2022) (ClinicalTrials.gov: NCT05789199 [Registration date: 16 February 2023]). Cefiderocol was made available prior to marketing authorisation in the compassionate use programme for those patients who had no alternative active treatment option, due to resistance or potential adverse events, for life-threatening Gram-negative bacterial infections; local institutional review board approval and patient consent were required for each eligible patient, for whom cefiderocol was requested. Following marketing authorisation by the European Medicines Agency, the same eligibility criteria were applicable in the EAP. Patients with *Acinetobacter* spp. in the EAP had been enrolled into the ARES study between April 2020 and April 2021 [25], therefore, were excluded from this study.

### Eligibility criteria

Adult hospitalised patients were eligible for inclusion in the PERSEUS study if they received cefiderocol continuously for at least 72 h for the first time for a confirmed aerobic Gram-negative bacterial infection, except *Acinetobacter* spp., at any infection site. The origin of the primary Gram-negative bacterial species for which cefiderocol was administered was cross-checked between the primary infection site and the biospecimen reported in the patient charts. If no confirmation was possible to make in cases of a mismatch, the patients were excluded from the analysis.

Patients were also excluded if they had any of the following criteria: enrolled into a clinical study of another investigational product; their medical records were incomplete for essential data, including cefiderocol dose, treatment duration, dosing schedule, or clinical outcome; or had a co-infection with confirmed cefiderocol-resistant Gram-negative bacteria in the prior 28 days of initiation of cefiderocol treatment for the current infection.

### Outcomes, variables and definitions

The main outcomes of the study were clinical cure, which was defined as cessation of cefiderocol treatment due to resolution of clinical signs and symptoms of the infection, and all-cause mortality (or survival) at Day 28 from the start of cefiderocol treatment in the primary analysis population. The primary aim of the study was to assess these outcomes in patients who were treated with cefiderocol for at least 72 h and up to 28 days. Clinical success was defined as a composite outcome of clinical cure or survival at Day 28. This outcome aimed to capture a favourable response for patients treated for maximum 28 days within the context of the early access programme when cefiderocol was requested for the treatment on an individual basis. Patients who died during therapy without assessment of any clinical response were considered as clinical failures. Patients with treatment duration > 28 days were excluded from this primary analysis to eliminate a survival bias.

The main variables collected were baseline demographics (age, sex) and clinical characteristics (admission type and diagnosis, comorbidities, Charlson Comorbidity Index [CCI], infection severity [APACHE II and SOFA scores], creatinine clearance) at the time of cefiderocol administration, the clinical and microbiological characteristics of the eligible Gram-negative bacterial infection (primary infection site, secondary bloodstream infection [for patients who had a known primary infection site], infection type [monomicrobial or polymicrobial infection with other Gram-negative species], Gram-negative species, antibiotic susceptibility profile), prior antibiotics (number of courses and duration), concomitant antibiotics, hospitalisation characteristics (ICU admission, mechanical ventilation, organ support type), cefiderocol use (duration of treatment, adverse drug reactions and relevant interventions) and discharge status. For patients with polymicrobial Gram-negative infections, the treating physician confirmed the primary pathogen for which cefiderocol was requested. Timing of cefiderocol treatment was represented by duration of prior antibiotic treatment. First-line cefiderocol treatment was defined as no prior antibiotic treatment was administered for the current infection. Data on antibiotic susceptibility phenotype was based on reported information in the medical charts. Because cefiderocol susceptibility testing was not routinely available, confirmed cefiderocol susceptibility was not a prerequisite of eligibility in the study. However, patients with confirmed cefiderocol-resistant co-infecting pathogens recorded for the previous 28 days, based on an available susceptibility test result, were excluded.

Outcomes were analysed for overall and by baseline pathogen, baseline characteristics of patients, severity of illness, prior and concomitant Gram-negative antibiotics, antimicrobial resistance status, cefiderocol usage pattern; additionally, 14-day all-cause mortality overall and adverse drug reactions as reported by the physicians associated with cefiderocol treatment were analysed.

### Statistics

Data collection was conducted between 31 July 2022 and 16 June 2023. All centres that received cefiderocol in the EAP were approached for participation. The overall primary analysis population included eligible patients infected by all Gram-negative bacterial species, except *Acinetobacter* spp. Subgroup analyses were performed for patients infected by *P. aeruginosa*, *Pseudomonas* spp., *K. pneumoniae*, and Other Enterobacterales and presented in the current manuscript. Subgroup analyses conducted for patients infected by *Stenotrophomonas maltophilia*, and Other rare non-fermenters are presented in more detail in the accompanying manuscript.

For continuous parameters, median and interquartile ranges were determined. For categorical parameters, the frequency and percentages were provided. Missing data were not imputed.

Univariate and multivariate logistic regression analyses were performed to examine associations between baseline clinically relevant variables and clinical cure in an exploratory analysis, and presented with OR with 95% CI. The *P* value was determined by the Wald test. In the stepwise logistic regression analysis, a *P* value > 0.1 was used to identify variables that were not significant, and *P* < 0.05 was considered statistically significant in the final model. The exploratory variables included age, sex, CCI points, septic shock, creatinine clearance, renal replacement therapy, secondary bloodstream infection, previous colonisation, polymicrobial infection status, immunosuppression status, Covid-19 status, *P. aeruginosa*, number of courses and days of prior Gram-negative antibiotics, and mechanical ventilation at baseline.

Unadjusted Kaplan–Meier survival analysis from initiation of cefiderocol treatment for up to 28 days was conducted for patients stratified by duration of prior antibiotic treatment, and subgroups were compared with log-rank test.

All analyses were performed using SAS^®^ version 9.4 version (SAS Institute Inc., Cary, NC, USA).

## Results

### Patient disposition

**Fig. 1:**
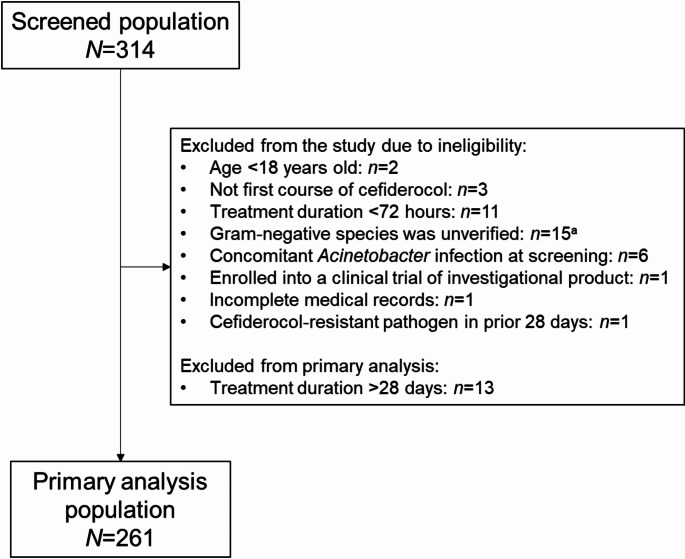
Patient enrolment and flow through the study; ^a^Verification between the infection site and the biospecimen was absent

The patient disposition is shown in Fig. 1. Of 314 screened patients in the EAP, 261 patients were eligible for inclusion in the primary analysis. Most of the exclusions from the study occurred due to lack of verified information on the baseline Gram-negative bacteria and treatment duration of < 72 h. A total of 13 patients were treated with cefiderocol for > 28 days, thus, excluded from the primary analysis. A total of 50 centres, mostly tertiary hospitals, participated in the study across Spain.

### Patient characteristics, comorbidities and baseline Gram-negative bacterial infections

The median age was 61.0 years and 77.4% were male (Table 1). At least one comorbid condition was present in 76.2% of patients, most frequently cancer, diabetes mellitus, chronic kidney disease and chronic obstructive pulmonary disease (Table S1). Cefiderocol was initiated in the ICU for > 60% of patients. At the time of cefiderocol initiation, the median SOFA score was 8.0, 47.1% of patients received mechanical ventilation, while septic shock (28.0%), renal replacement therapy (27.2%), creatinine clearance < 60 mL/min (30.5%), immunosuppression (30.3%) and symptomatic COVID-19 infection (24.1%) were common (Table 1).

**Table 1:** Patients’ baseline demographics and clinical characteristics, and resistance profile of baseline Gram-negative pathogens in the overall primary analysis population (*N* = 261) and in patients with *P. aeruginosa* (*N* = 174)

	Overall	*P*. aeruginosa
Overall, *n* (%)	261 (100)	174 (66.7)
Age (years), median (IQR)	61.0 (49.0–68.0)	61.0 (52.0–68.0)
Sex (male), *n* (%)	202 (77.4)	138 (79.3)
CCI score, median (IQR)	3.0 (2.0–4.0)	3.0 (2.0–5.0)
SOFA score, median (IQR)	8.0 (4.0–11.0)	7.0 (3.0–10.0)
APACHE, median (IQR)	15.0 (10.5–22.0)	15.5 (10.0–22.0)
ICU, *n* (%)	165 (63.2)	109 (62.6)
Mechanical ventilation at baseline, *n* (%)	123 (47.1)	79 (45.4)
Symptomatic COVID-19 during hospitalisation, *n* (%)	63 (24.1)	46 (26.4)
Septic shock, *n* (%)	73 (28.0)	47 (27.0)
ECMO, *n* (%)	12 (4.6)	8 (4.6)
RRT, *n* (%)	71 (27.2)	45 (25.9)
Creatinine clearance < 60 mL/min, *n* (%)^a^	54/177 (30.5)	37/119 (31.1)
Immunosuppressed, *n* (%)^b^	79 (30.3)	41 (23.6)
Transplant recipient, *n* (%)	54 (20.7)	23 (13.2)
Solid	33/54 (61.1)	17/23 (73.9)
Haematopoietic	21/54 (38.9)	6/23 (26.1)
Primary infection site, *n* (%)		
Respiratory	125 (47.9)	81 (46.6)
Urinary	38 (14.6)	28 (16.1)
Intra-abdominal	38 (14.6)	23 (13.2)
Skin and soft tissue	26 (10.0)	22 (12.6)
Bloodstream	24 (9.2)	11 (6.3)
Bloodstream (catheter related)	15 (5.7)	7 (4.0)
Bloodstream (unknown source)	9 (3.4)	4 (2.3)
Bone and joint	6 (2.3)	6 (3.4)
Other^c^	4 (1.5)	3 (1.7)
Secondary bloodstream infection, *n* (%)	45 (17.2)	28 (16.1)
Polymicrobial Gram-negative infection, *n* (%)^d^	51 (19.5)	36 (20.7)
Previous colonisation with the same infecting pathogen, *n/N’* (%)	135/255 (52.9)	89/171 (52.0)
Meropenem resistant, *n/N’* (%)^e^	189/206 (91.7)	139/147 (94.6)
Ceftazidime-avibactam resistant, *n/N’* (%)^f^	134/160 (83.8)	96/112 (85.7)
Ceftolozane-tazobactam resistant, *n/N’* (%)^f^	99/130 (76.2)	75/105 (71.4)
Resistant to ceftazidime-avibactam and ceftolozane-tazobactam, *n/N’* (%)^g^	99/129 (76.7)	74/103 (71.8)

*N*’, number of patients with available information

CCI, Charlson Comorbidity Index; COVID-19, coronavirus disease-2019; ECMO, extracorporeal membrane oxygenation; RRT, renal replacement therapy

^a^Excluding patients on RRT; denominator excludes missing data

^b^Transplant recipient, immunosuppressive treatment (e.g. high-dose corticosteroids, calcineurin inhibitors, anti-CD20, IL-1 inhibitors and IL-6 inhibitors)

^c^Includes central nervous system infection (*n* = 2), mediastinitis (*n* = 2) in overall

^d^Primary pathogen in polymicrobial infections, for which cefiderocol was requested, was confirmed by the treating physician

^e^Susceptibility test results were reported at local site

^g^Includes patients with susceptibility test results for both ceftazidime-avibactam and ceftolozane-tazobactam

*P. aeruginosa* was the most frequent Gram-negative pathogen (174/261; 66.7%) (Table 1). The current manuscript reports on patients with *P. aeruginosa* (*N* = 174), *Pseudomonas* spp. (*N* = 15), *K. pneumoniae* (*N* = 26) and other Enterobacterales (*N* = 12). Further demographic, baseline characteristics and other data for patients infected by *S. maltophilia* (*N* = 20), *Burkholderia cepacia* complex (*N* = 8), *Achromobacter* spp. (*N* = 5) and *Ralstonia mannitolilytica* (*N* = 1) are summarised in the accompanying paper.

Baseline demographics and clinical characteristics for patients infected by *P. aeruginosa* were similar to those in the overall population (Table 1, Table S1, Table S2). There were some numerical differences in baseline demographics and clinical characteristics among patients infected by *Pseudomonas* spp., *K. pneumoniae* and other Enterobacterales (Table S1, Table S3).

The most frequent infection site was the respiratory tract overall, and among patients with *P. aeruginosa* and *K. pneumoniae* infections (Table 1, Table S3). While > 50% of the population were previously colonised with the same pathogen, particularly among patients with Enterobacterales and *K. pneumoniae*, secondary bloodstream infection and polymicrobial infections were less frequent (Table 1, Table S3).

Based on susceptibility data reported in the medical charts, > 90% of isolates with confirmed susceptibility status were resistant to meropenem, 83.8% were resistant to ceftazidime-avibactam and 76.2% were resistant to ceftolozane-tazobactam (Table 1). The mechanisms of β-lactam resistance were reported for 48.7% of isolates overall (*n* = 127) and 46.0% of *P. aeruginosa* isolates (*n* = 80); metallo-β-lactamases, mainly imipenemase and Verona integron-encoded carbapenemases, were most frequent enzymes (overall: *n* = 98; *P. aeruginosa*: *n* = 73) (Table S2).

Patient characteristics by infection site are shown in Table S4. Patients with urinary tract infection were older (median age 66.5 years) than patients with any other infection site (median age range 54.5–62 years). The Charlson Comorbidity Index was highest (4.0) among patients with intra-abdominal infection, urinary tract infection, and bone and joint infection. Polymicrobial infections were most frequent in patients with respiratory tract infections (24.0%) and other infection site (25.0%) (Table S4).

### Treatment patterns

Prior Gram-negative antibiotics were administered to 81.2% of patients (missing: *n* = 7). The median number of prior courses of antibiotic treatments was 2.0 and the median duration of prior antibiotic treatment was 6.0 days (Table 2). Patients most frequently received colistin (36.4%), ceftazidime-avibactam (31.0%), meropenem (30.7%); prior antibiotics varied by baseline Gram-negative pathogen (Table S5).

**Table 2:** Pattern of cefiderocol use, and prior and concomitant antibiotic use in the overall primary analysis population (*N* = 261) and in patients with *P. aeruginosa* (*N* = 174)

Prior antibiotic and cefiderocol use	Overall *N* = 261	*P*. aeruginosa *N* = 174
Prior antibiotics, *n* (%)^a^	212 (81.2)	147 (84.5)
Number of prior courses of Gram-negative antibiotic treatments, median (IQR)^b^	2.0 (1.0–3.0)	2.0 (1.0–4.0)
1, *n* (%)	54 (25.5)	37 (25.2)
2, *n* (%)	62 (29.2)	43 (29.3)
≥ 3, *n* (%)	96 (45.3)	67 (45.6)
None, *n (%)*	42 (16.1)	25 (14.4)
Unknown, *n*	7	2
Duration of prior Gram-negative antibiotic treatment (days), median (IQR)	6 (3.0–10.1)	5.5 (3.0–9.5)
≤ 3, *n* (%)	55/212 (25.9)	46/147 (31.3)
4–7, *n* (%)	70/212 (33.0)	47/147 (32.0)
> 7, *n* (%)	87/212 (41.0)	54/147 (36.7)
Rationale for administration of cefiderocol, *n* (%)^c^		
Resistance to all tested antibiotics	169 (64.8)	116 (66.7)
Treatment failure of prior antibiotics	116 (44.4)	75 (43.1)
Adverse events to other susceptible antibiotics	21 (8.0)	14 (8.0)
Other	26 (10.0)	16 (9.2)
Cefiderocol as first-line therapy, *n* (%)	42 (16.1)	25 (14.4)
Duration of cefiderocol treatment (days), median (IQR)	10.0 (7.0–14.0)	11.0 (7.0–15.0)
Combination therapy given with cefiderocol, *n* (%)^d^	91 (34.9)	56 (32.2)
Number of antibiotics used concomitantly with cefiderocol, *n/N’* (%)	*N’*=91	*N'* = 56
1	41 (15.7)	25 (14.4)
2	25 (9.6)	16 (9.2)
≥ 3	25 (9.6)	15 (8.6)
Cefiderocol dosing, *n* (%)		
Every 4 h	3 (1.1)	2 (1.1)
Every 6 h	25 (9.6)	17 (9.8)
Every 8 h	213 (81.6)	143 (82.2)
Every 12 h	20 (7.7)	12 (6.9)
Other	0 (0)	0 (0)

*N*’, Number of patients with available information

^a^Prior antibiotics were administered to a total of 219 patients overall; data are shown for 212 patients with full data set; information was missing for 7 patients

^b^List of prior antibiotics is included in Table S5

^c^Investigators could select more than one options

^d^Includes antibiotics with Gram-negative coverage that have been started before, concomitantly or during the same treatment period

Physicians most frequently administered cefiderocol due to resistance to all other tested antibiotics (64.8%) and/or failure of prior treatment (44.4%) (Table 2). Cefiderocol was administered as first-line treatment for 16.1% of patients overall (*n* = 42) and 14.4% of patients with *P. aeruginosa* (*N* = 25), while the median duration of cefiderocol treatment was 10.0 days and 11.0 days, respectively. The median duration of cefiderocol treatment by infection site was longer in patients with skin and skin structure infections (12.5 days), bone and joint infections (16.5 days), and other infection sites (18.0 days) (Table S4). Among patients with respiratory tract infections, nearly half (46.4%) of patients received cefiderocol combination therapy and 53.0% of patients received cefiderocol following administration of ≥ 3 prior courses of antibiotic treatments (Table S4). Cefiderocol was administered every 8 h to 213 (81.6%) patients overall. Only one patient, infected by *K. oxytoca*, discontinued cefiderocol treatment due to development of on-therapy resistance. Cefiderocol was given in combination therapy with antibiotics with Gram-negative coverage to approximately one-third of patients overall and of those with *P. aeruginosa*. Colistin was the antibiotic most frequently co-administered with cefiderocol in 19.5% of the patients (Table S6).

### Outcomes overall and by baseline infection variables

**Fig. 2:**
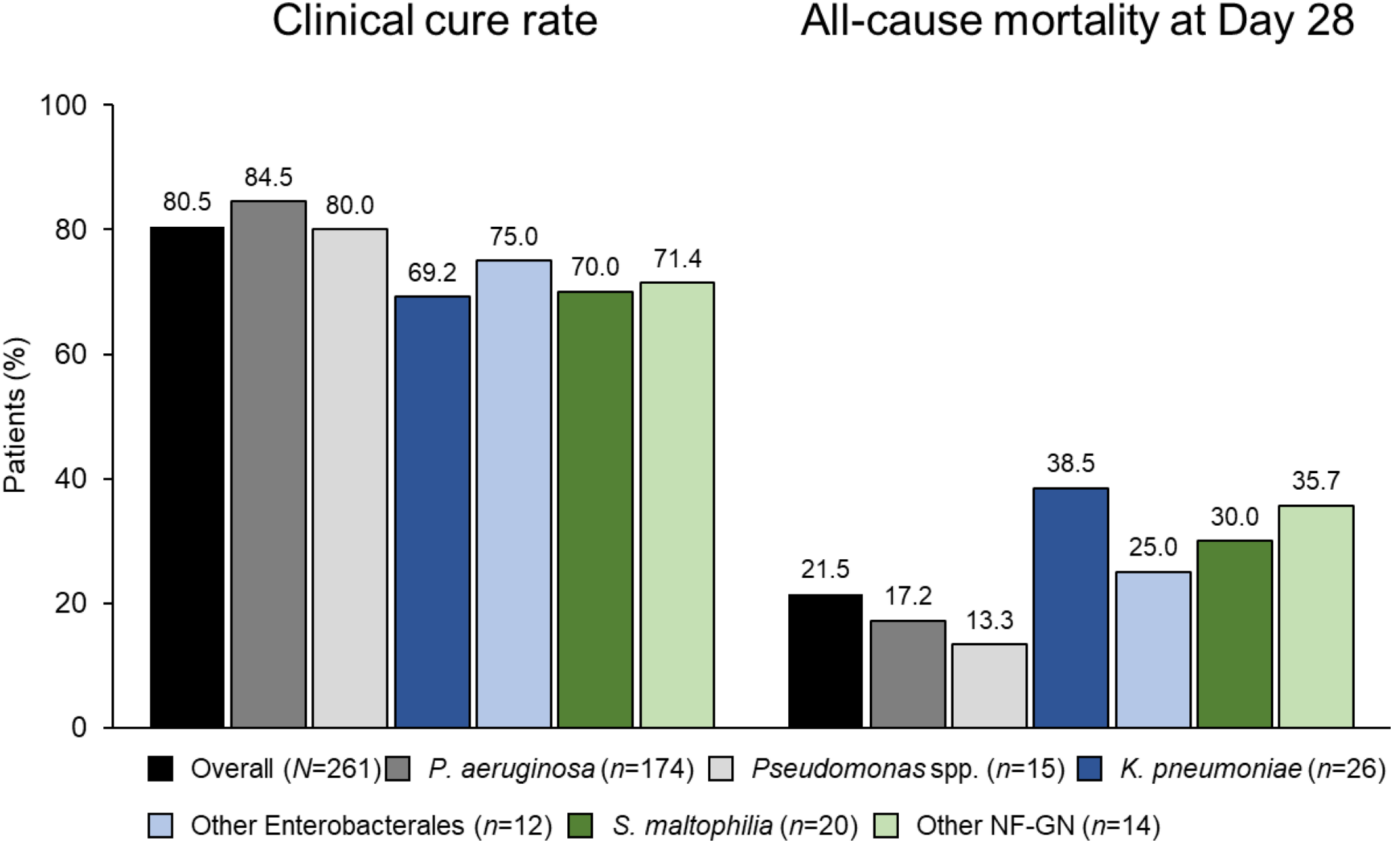
Rates of clinical cure at end of treatment and all-cause mortality at Day 28, in the overall primary analysis population (*N* = 261) and by baseline Gram-negative pathogen. NF-GN, non-fermenter Gram-negative; Other Enterobacterales (*n*): *S. marcescens* (5), *E. cloacae* (3), *K. oxytoca* (2), *C. freundii* (1), *Serratia* spp. (1); *Pseudomonas* spp. (*n*): *P. putida* (12), *Pseudomonas fluorescens/putida* group (1), *P. fluorescens* (1), *P. nitroreducens* (1); Other NF-GN (*n*): *Burkholderia cepacia* complex (8), *Achromobacter* spp. (5), *Ralstonia mannitolilytica* (1)

In the primary analysis population, 80.5% of patients (210/261) had clinical cure at end of treatment and 28-day all-cause mortality was 21.5% (56/261) (Fig. 2). In the subset of patients with *P. aeruginosa*, the clinical cure rate was 84.5% (147/174), and the 28-day mortality rate was 17.2% (30/174) (Fig. 2). All-cause mortality at day 14 was 14.2% (37/261) in the overall primary analysis population and 10.3% (18/174) for patients with *P. aeruginosa* infections. The overall clinical success rate was 84.3% (220/261) and 89.1% (155/174) in patients with *P. aeruginosa*.

**Fig. 3:**
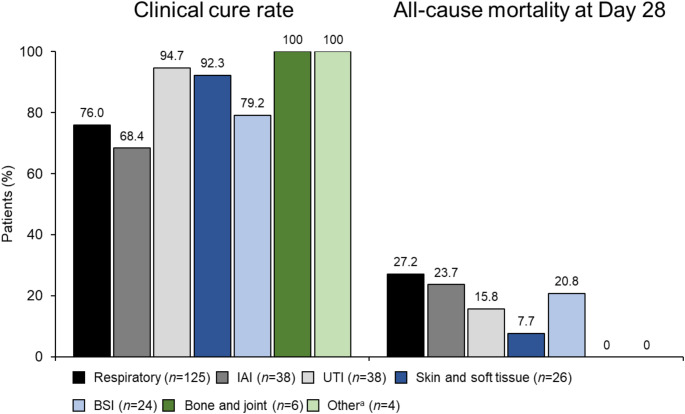
Rates of clinical cure at end of treatment and all-cause mortality at Day 28 by infection site in the overall primary analysis population (*N* = 261). BSI, bloodstream infection; IAI, intra-abdominal infection; UTI, urinary tract infection; ^a^Includes central nervous system infection (*n* = 2) and mediastinitis (*n* = 2)

Clinical cure rates varied by infection site; thus, the highest clinical cure rates were found among patients with infections of the urinary tract, skin and soft tissue, bone and joint, and other sites (Fig. 3). Rates of clinical cure and 28-day all-cause mortality were similar for patients with respiratory tract infections, intra-abdominal infections, and bloodstream infections overall (Fig. 3).

**Fig. 4:**
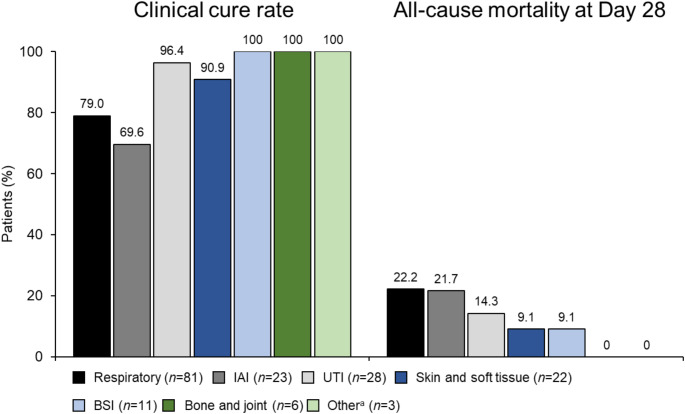
Rates of clinical cure at end of treatment and all-cause mortality at Day 28 by infection site in patients with *Pseudomonas aeruginosa* (*N* = 174). BSI, bloodstream infection; IAI, intra-abdominal infection; UTI, urinary tract infection; ^a^Includes central nervous system infection (*n* = 2) and mediastinitis (*n* = 1)

Similar trends were observed among patients with *P. aeruginosa* infections (Fig. 4).

The presence or absence of immunosuppression, COVID-19, colonisation with the same multidrug-resistant pathogen, mono- or polymicrobial infection had no effect on clinical cure and 28-day mortality rates (Table S7). However, presence of septic shock, ICU admission, renal replacement therapy and mechanical ventilation at baseline resulted in lower clinical cure rates and higher all-cause mortality rates than in patients without these features (Table S7). Rates of clinical cure and 28-day all-cause mortality by pathogen and infection site are shown in Table S8.

### Outcomes by antibiotic resistance, prior antibiotic use and Cefiderocol use

Clinical cure and 28-day all-cause mortality rates were similar between subgroups of infections caused by pathogens resistant to meropenem, ceftolozane-tazobactam and/or ceftazidime-avibactam, or colistin (Table 3, Table S7).

**Table 3:** Clinical cure, all-cause mortality at day 28, and composite clinical success rates overall, by antibiotic resistance and antibiotic use in the overall primary analysis population

Baseline characteristics	Overall	Clinical cure at EOT	All-cause mortality at Day 28	Composite clinical success
Overall, *n/N* (%)	261 (100)	210 (80.5)	56 (21.5)	220 (84.3)
Meropenem resistance	*N’*=206			
Yes, *n/N’* (%)	189 (91.7)	152 (80.4)	39 (20.6)	160 (84.7)
Ceftolozane-tazobactam resistance	*N’*=130			
Yes, *n/N’* (%)	99 (76.2)	82 (82.8)	17 (17.2)	85 (85.9)
Ceftazidime-avibactam resistance	*N’*=160			
Yes, *n/N’* (%)	134 (83.8)	107 (79.9)	31 (23.1)	111 (82.8)
Cross-resistance to ceftazidime-avibactam and ceftolozane-tazobactam	*N’*=129			
Yes, *n/N’* (%)	99 (76.7)	82 (82.9)	17 (17.2)	85 (85.9)
Colistin resistance	*N*’=210			
Yes, *n/N’* (%)	35 (16.7)	28 (80.0)	8 (22.9)	28 (80.0)
Number of days with prior antibiotics, *n/N’* (%)	*N’*=212	*N* = 168	*N* = 47	*N* = 178
≤ 3	55 (25.9)	49 (89.1)	9 (16.4)	49 (89.1)
4–7	70 (33.0)	59 (84.3)	13 (18.6)	62 (88.6)
> 7	87 (41.0)	60 (69.0)	25 (28.7)	67 (77.0)
Cefiderocol as first line, *n* (%)^a^				
No	219 (83.9)	172 (78.5)	50 (22.8)	182 (83.1)
Yes	42 (16.1)	38 (90.5)	6 (14.3)	38 (90.5)
Combination treatment, *n* (%)				
No	170 (65.1)	143 (84.1)	30 (17.6)	150 (88.2)
Yes	91 (34.9)	67 (73.6)	26 (28.6)	70 (76.9)

*N*’, total number of patients in the category

EOT, end of treatment

^a^Patients with first-line treatment received no prior antibiotic treatment for the current infection

Among patients who received colistin, meropenem, ceftazidime-avibactam, or ceftolozane-tazobactam prior to cefiderocol, the clinical cure rates ranged between 72.5% and 77.9% overall, and between 78.2% and 81.5% for patients with *P. aeruginosa* infections (Table S7). Corresponding 28-day all-cause mortality rates ranged between 16.7% and 28.8%, and between 11.1% and 21.8% by prior antibiotic treatment, respectively (Table S7).

Cefiderocol administered as first-line therapy resulted in numerically higher clinical cure rates compared with patients who received it later (Table 3). Patients with cefiderocol combination therapy had numerically lower rate of clinical cure compared with monotherapy (Table 3, Table S7).

### Univariate and multivariate regression analysis and Kaplan-Meier survival analysis

Among the risk factors identified in the univariate and multivariate stepwise logistic regression analyses for clinical cure, duration of prior antibiotic treatment before cefiderocol initiation (i.e. >7 days vs. 0 day [categorical variable]) (OR 0.19, 95% CI 0.05–0.56) and receipt of mechanical ventilation at baseline (OR 0.32, 95% CI 0.15–0.67) were significantly associated with a reduced probability of achieving clinical cure (Table 4). The only variable included in the final model showing a trend towards better outcome was infection by *P. aeruginosa* (OR 1.97, 95% CI 0.97–4.00). Among patients with *P. aeruginosa*, presence of septic shock (OR 0.22, 95% CI 0.08–0.58), receipt of mechanical ventilation at baseline (OR 0.35, 95% CI 0.11–0.98), duration of prior antibiotic treatment (OR 0.93, 95% CI 0.87–0.98 [continuous variable]) and age (OR 0.94, 95% CI 0.90–0.99) showed significant effect on clinical cure (Table S9).

**Table 4:** Univariate and multivariate logistic regression analysis for clinical cure at EOT in the overall primary analysis population (*N* = 261)

Clinical cure at EOT	Univariate analysis (*N* = 261)			Multivariate analysis (*N* = 254)		
	OR	95% CI	Wald test *P* value	OR	95% CI	Wald test *P* value
Age (continuous)	0.99	0.96–1.01	0.2394			
Age (categorical)^a^	0.73	0.28–1.88	0.5135			
Sex (male vs. female)	0.80	0.36–1.66	0.5689			
CCI score (continuous)	0.90	0.78–1.03	0.1085	0.87	0.75–1.01	0.0728
Septic shock (yes vs. no)	0.38	0.20–0.72	0.0029	0.50	0.24–1.07	0.0719
Creatinine clearance (> 30 mL/min vs. ≤ 30 mL/min)	0.59	0.19–1.49	0.3055			
RRT (yes vs. no)	0.44	0.09–3.19	0.3426			
Secondary bloodstream infection (yes vs. no)	0.68	0.32–1.53	0.3337			
Previously colonised (yes vs. no)	1.10	0.59–2.06	0.7644			
Infection (polymicrobial vs. monomicrobial)	0.74	0.36–1.60	0.4243			
Immunosuppression (yes vs. no)	0.75	0.40–1.45	0.3847			
COVID-19 (yes vs. no)	0.80	0.41–1.65	0.5381			
*Pseudomonas aeruginosa* (yes vs. no)	2.07	1.11–3.88	0.0220	1.97	0.97–4.00	0.0600
Prior antibiotics (yes vs. no)	0.39	0.11–1.02	0.0833			
Duration of prior GN antibiotic (continuous)	0.96	0.93–0.99	0.0231			
Duration of prior GN antibiotic (categorical)^b^	0.57	0.40–0.78	0.0010	0.59	0.41–0.82	0.0025
Duration of prior GN antibiotic (1–3 days vs. 0 days)				0.45	0.10–1.82	0.2693
Duration of prior GN antibiotic (4–7 days vs. 0 days)				0.48	0.12–1.60	0.2561
Duration of prior GN antibiotic (> 7 days vs. 0 days)				0.19	0.05–0.56	0.0054
Mechanical ventilation at baseline (yes vs. no)	0.29	0.15–0.56	0.0003	0.32	0.15–0.67	0.0032
Mortality (> 20% vs. < 20%)	0.17	0.06–0.49	0.0011			

CCI, Charlson Comorbidity Index; COVID-19, coronavirus disease-2019; EOT, end of treatment; GN, Gram-negative; RRT, renal replacement therapy

^a^1: <50 years; 2: 50–59 years; 3: 60–69 years; 4: 70–79 years; 5: ≥80 years

^b^1: 0 days; 2: 1–3 days; 3: 4–7 days; 4: >7 days

**Fig. 5:**
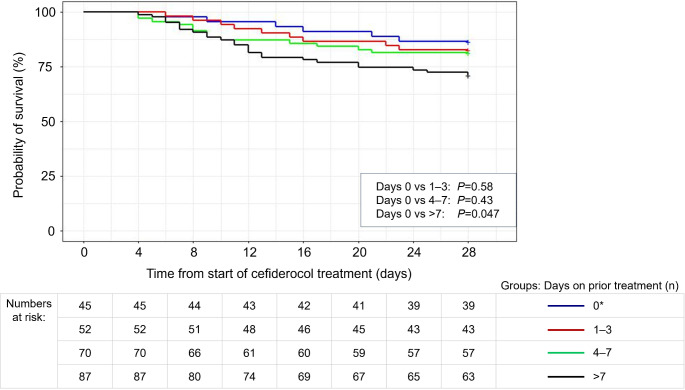
Kaplan–Meier analysis of probability of survival from initiation of cefiderocol treatment for up to 28 days in the overall primary analysis population (*N* = 261; missing *n* = 7) *This category includes three patients who did not receive at least one full calendar day of prior antibiotic treatment but received one or two doses

To evaluate the impact of duration of prior antibiotic treatment on survival from initiation of cefiderocol treatment for up to 28 days, an unadjusted Kaplan-Meier survival analysis was performed. The analysis showed that the probability of survival was significantly lower when patients received prior antibiotic treatment for longer than 7 days compared with patients who received no prior antibiotic treatment (Fig. 5).

### Adverse drug reactions

Seven patients (2.2%) experienced adverse drug reactions, three of which were serious and resulted in discontinuation of treatment (Table 5). Six patients recovered and one patient died with a serious event (i.e. toxic epidermal necrolysis).

**Table 5:** Adverse drug reactions in all screened patients

Adverse drug reactions, *n* (%)	Overall *N* = 314^a^
Any	7 (2.2)
Discontinuation due to ADR^b^	3 (1.0)
Serious ADR	3 (1.0)
Discontinuation due to serious ADR^b^	3 (1.0)
Death due to serious ADR^c^	1 (0.3)

ADR, adverse drug reaction

^a^Number of screened patients

^b^One patient discontinued due to septic rash (patient was withdrawn from the primary analysis population because of cefiderocol treatment < 72 h); one patient discontinued due to mild leucopoenia; one patient discontinued due to fatal toxic epidermal necrolysis

^c^One event of fatal toxic epidermal necrolysis

## Discussion

The results of the PERSEUS study highlighted the pattern of cefiderocol use, and the effectiveness of cefiderocol in aerobic Gram-negative infections, except *A. baumannii*, in the EAP in Spain. The study population was severely ill, as evidenced by high SOFA score, frequent ICU admission and reliance on organ support at baseline, presence of septic shock, secondary bloodstream infection and high-risk comorbidities (e.g. cancer, transplant and immunosuppression), as well as treatment failure with prior antibiotics. At baseline, most patients (> 85%) were infected by non-fermenting Gram-negative bacteria and over half of the population had documented prior colonisation by the same Gram-negative bacterial species. Cefiderocol, administered for a median of 10 days, was well tolerated in this population and adverse drug reactions were reported infrequently.

Because cefiderocol was requested by the treating physicians to be administered to patients with limited treatment options, resolution of clinical signs and symptoms at end of cefiderocol treatment is an important outcome as well as survival. It was found that cefiderocol treatment resulted in a high clinical cure rate (80.5%) at end of treatment and 21.5% of patients died by Day 28. Among patients infected by *P. aeruginosa*, clinical cure rate was numerically higher (84.5%) with a mortality rate of 17.2%. Similar to previous studies [26, 27, 28], in the PERSEUS study *P. aeruginosa* was the most frequently isolated pathogen in respiratory tract infections and outcomes were similar to that of the overall cohort and were within the range of previous studies of MDR, CR or XDR *P. aeruginosa* pneumonia and other infections [26, 28, 29, 30, 31, 32]. In an early retrospective study of compassionate use of cefiderocol in patients with a variety of infections caused by XDR or DTR *P. aeruginosa*, 70.6% and 76.5% of patients had clinical and microbiological cure, respectively, and day-30 mortality was 23.5% [23].

The most recent retrospective matched comparative study (CACTUS) between ceftolozane-tazobactam and ceftazidime-avibactam in the treatment of severe pneumonia and bacteraemia caused by *P. aeruginosa* showed overall clinical success rates of 61% and 52%, and 30-day mortality rates of 23% and 24%, respectively [33]. Because the definition of clinical success in the CACTUS study was more stringent, comparison with the results of the PERSEUS study is not plausible, but all-cause mortality rate in the PERSEUS study in patients with *P. aeruginosa* infections was numerically lower than in CACTUS [33]. It is also noteworthy that the median time to initiation of ceftolozane-tazobactam and ceftazidime-avibactam were 71.8 h and 70.9 h, respectively, in the CACTUS study [33], while in the PERSEUS study, prior antibiotics were administered for a median duration of 5.5 days in patients with *P. aeruginosa* infections. The outcomes of the PERSEUS study in patients infected by CR *P. aeruginosa* isolates resistant to the newer β-lactam–β-lactamase inhibitors compare favourably with recent real-world observational studies of ceftolozane-tazobactam (18.8–23% mortality rates) or ceftazidime-avibactam (13.3–28.2% mortality rates) in infections caused by *P. aeruginosa* isolates susceptible to these agents [26, 28, 33, 34, 35], given the severity of illness, multiple lines of prior antibiotic therapy, and thus a longer time to administration of effective antibiotic therapy seen in this EAP population.

Cefiderocol has demonstrated, both globally and in Spain, excellent susceptibility rates (90–100%) for *P. aeruginosa*, including CR isolates or isolates that are resistant to ceftazidime-avibactam and/or ceftolozane-tazobactam [15, 16, 18, 19, 20, 21, 36]. Compared with various anti-pseudomonal agents, cefiderocol showed greater in vitro activity against CR *P. aeruginosa* isolates (with MIC_50/90_ of 0.5/2 mg/L) carrying serine- and metallo-β-lactamases, particularly against imipenemase and Verona integron-encoded carbapenemase-producing *P. aeruginosa* [19, 36]. In this study, > 40% of *P. aeruginosa* isolates carried a metallo-β-lactamase enzyme. Thus, the current clinical data from this study, supported by the high level of in vitro activity, highlight cefiderocol as a promising anti-pseudomonal agent.

Delay in appropriate antibiotic treatment in critically ill patients adversely affects clinical outcomes and increases the risk of mortality [37, 38]. In this EAP cohort, delays or variations in time to initiate cefiderocol treatment may have contributed to the overall outcomes. There was a significant association in multivariate regression analysis towards a decreasing probability of achieving clinical cure with longer duration of prior antibiotic treatment and receipt of mechanical ventilation. Accordingly, higher clinical cure and lower all-cause mortality rates were found with no or shorter prior antibiotic courses in the overall population. Importantly, among patients who received cefiderocol as first-line therapy, the clinical cure rate was higher than among those patients receiving cefiderocol as later-line therapy, and this was particularly significant in infections caused by *P. aeruginosa*. The Kaplan–Meier survival analysis in our study showed that prior treatment for > 7 days may increase the risk of mortality, although this analysis was not adjusted for confounders. Furthermore, cefiderocol treatment was equally effective regardless of the prior antibiotic agent. These data, along with the high rate of cefiderocol susceptibility [16, 18, 39], suggest that for ‘at-risk’ patients cefiderocol represents a valuable early treatment for severe infections.

Current guidelines suggest that cefiderocol should be reserved for the treatment of CR Enterobacterales, CR *A. baumannii*, DTR *P. aeruginosa* and/or *S. maltophilia*, who experience treatment failure of other first-line and/or second-line therapy, and that known resistance profile of the Gram-negative pathogen should support the selection of this antibiotic [10, 40, 41, 42]. Almost all baseline isolates were resistant to carbapenems, ceftazidime-avibactam and ceftolozane-tazobactam or both. Clinical cure rates were similar regardless of the resistance profile of the baseline Gram-negative pathogens, and a proportion of those patients also experienced prior treatment failure. Nevertheless, the results of this study suggest that earlier treatment (i.e. first-line therapy) with cefiderocol rather than rescue therapy is associated with improved clinical outcomes in patients with MDR and CR Gram-negative infections.

Cefiderocol was initiated as monotherapy for 65% of patients. Real-world data suggest that both cefiderocol monotherapy and combination therapy are similarly effective, although clinical judgement in administering cefiderocol in monotherapy may be influenced by the severity of infection and the underlying illness [25, 27, 43, 44, 45, 46]. In the current study, cefiderocol clinical cure rates were numerically higher among patients receiving monotherapy versus those receiving combination therapy (Table 3, Table S7). Cefiderocol combination therapy was most frequent in patients with respiratory tract, intra-abdominal, bone and joint, and other infection sites (Table S4), and in those with rare non-fermenters [47]. Patients with combination therapy were more frequently in the ICU and received organ supportive care, suggesting more severe illness in these patients (*data not shown*).

The main limitation of the study was that it was a non-comparative retrospective observational study; thus, results should be considered as descriptive and confirmed in prospective studies. Additionally, the number of patients in certain subgroups was small. As cefiderocol was used through the EAP on a patient-by-patient basis, patient characteristics, clinical diagnoses and access to medication may not precisely reflect the use of cefiderocol following regular hospital prescriptions in routine clinical practice. Other limitations include the very limited number of follow-up microbiological samples; thus, investigation of recurrent infections was not feasible in this study; microbiological results from the available baseline samples will be published elsewhere. Susceptibility information was based on reports in the medical charts without confirmatory testing by a central laboratory. Cefiderocol susceptibility test was not routinely available in clinical microbiology laboratories because susceptibility testing methods were not yet approved during the study period and it was not prerequisite to be enrolled into this study. Future investigations are required to collect accurate microbiological information on susceptibility and mechanisms of resistance for the available isolates.

## Conclusions

The results of this study support the effectiveness and activity of cefiderocol treatment in a high clinical acuity patient population infected by a variety of Gram-negative bacterial species, including non-fermenters and Enterobacterales, for which treatment options are limited. High clinical cure rates were observed in patients with prior treatment failure and/or resistance to current standard-of-care antibiotics regardless of the infecting pathogen or site of infection. A significant association with clinical cure was found when cefiderocol was administered early, thus, avoiding delay of an appropriate antibiotic treatment with potent in vitro activity. Cefiderocol was well tolerated in this critically ill population. The data corroborate the value of cefiderocol as an appropriate antibiotic treatment choice for challenging Gram-negative bacterial infections with limited treatment options, particularly for *P. aeruginosa*.

## Electronic supplementary material

Below is the link to the electronic supplementary material.

Supplementary Material 1

## Data Availability

Data analysed in the current study are not in the public domain. However, Shionogi is committed to share data with investigators and researchers at reasonable requests. Shionogi’s policy on sharing clinical trial data can be found at: https://www.shionogi.com/global/en/company/policies/shionogi-group-clinical-trial-data-transparency-policy.html.
