# Supplementary material for: Effectiveness and safety of cefiderocol treatment in patients with Gram-negative bacterial infections in Spain in the early access programme: results of the PERSEUS study
Source: Eur J Clin Microbiol Infect Dis. 2025 Mar 25;44(6):1375–90. doi: 10.1007/s10096-025-05108-6 (PMC12116923; doi:10.1007/s10096-025-05108-6)
Supplement: Supplementary file 1 — Supplementary Material 1 [file 10096_2025_5108_MOESM1_ESM.docx]

**Title**

Effectiveness and safety of cefiderocol treatment in patients with Gram-negative bacterial infections in Spain in the early access programme: results of the PERSEUS study

**Authors**

Julian Torre-Cisneros^1,2,3,4^ · Benito Almirante^2,5^ · Carmen De La Fuente Martos^1,2,6^ · Pedro Rascado^7^ · Miguel Salavert Lletí^8^ · Miguel Sánchez-García^9^ · Alex Soriano^2,10,11^ · Maria Cruz Soriano-Cuesta^12^ · A. Javier Gonzalez Calvo^13^ · Andreas Karas^14^ · Jessica Sarda^13^ · Stefano Verardi^14^ · Ricard Ferrer^15^

**Affiliations**

^1^Maimónides Biomedical Research Institute of Córdoba (IMIBIC), Reina Sofia University Hospital of Córdoba, Córdoba, Spain;

^2^Centro de Investigación Biomédica en Red de Enfermedades Infecciosas, Instituto de Salud Carlos III, Madrid, Spain;

^3^Infectious Diseases Service, Reina Sofia University Hospital of Córdoba, Córdoba, Spain;

^4^Department of Medical and Surgical Sciences, University of Córdoba, Córdoba, Spain;

^5^Infectious Diseases Department, Hospital Universitario Vall d' Hebrón, Barcelona, Spain;

^6^Critical Care Service, Hospital Universitario Reina Sofía, Córdoba, Spain;

^7^Intensive Care Unit, Complejo Hospitalario Universitario Santiago de Compostela, Santiago de Compostela, Spain;

^8^Infectious Diseases Unit, Medical Clinic Department, Hospital Universitario y Politécnico La Fe, Valencia, Spain;

^9^Critical Care Department, Hospital Clínico San Carlos, Universidad Complutense, Madrid, Spain;

^10^Department of Infectious Diseases, University of Barcelona, Hospital Clinic of Barcelona, Barcelona, Spain;

^11^IDIBAPS, Institut d'Investigacions Biomèdiques Agustí-Pi Sunyer, Barcelona, Spain;

^12^Intensive Care Medicine Department, Hospital Universitario Ramón y Cajal, Madrid, Spain;

^13^Shionogi SLU, Madrid, Spain;

^14^Shionogi BV, London, United Kingdom;

^15^Intensive Care Department, Hospital Universitari Vall d’Hebrón, SODIR Reseach Group, Vall d’Hebron Institut de Recerca, Universitat Autònoma de Barcelona, Barcelona, Spain.

**Corresponding author**

Jessica Sarda

Shionogi SLU, Calle de Serrano 45, Madrid, 28001, Spain

Email: jessica.sarda@shionogi.eu

Tel: +34 600 866 774

**Table S1.** Predisposing conditions of patients, overall and by type of Gram-negative bacteria in the overall primary analysis population (*N*=261) and in patients with *P. aeruginosa* (*N*=174), *Pseudomonas* spp. (*N*=15), *K. pneumoniae* (*N*=26), and Other Enterobacterales (*N*=12)

| Medical history, *n* (%) | Overall | *P. aeruginosa* | *Pseudomonas* spp.^a^ | *K. pneumoniae* | Other Enterobacterales^b^ |
| --- | --- | --- | --- | --- | --- |
|  | *N*=261 | *N*=174 | *N*=15 | *N*=26 | *N*=12 |
| Any pre-existing medical condition | 199 (76.2) | 135 (77.6) | 13 (86.7) | 18 (69.2) | 7 (58.3) |
| Tumour (solid organ or haematological) within the last 5 years | 62 (23.8) | 35 (20.1) | 11 (73.3) | 3 (11.5) | 3 (25.0) |
| Diabetes mellitus | 58 (22.2) | 45 (25.9) | 1 (6.7) | 8 (30.8) | 0 (0) |
| Moderate or severe chronic renal disease | 34 (13.0) | 24 (13.8) | 0 (0) | 5 (19.2) | 1 (8.3) |
| Chronic obstructive pulmonary disease | 27 (10.3) | 21 (12.1) | 0 (0) | 3 (11.5) | 1 (8.3) |
| Peripheral vascular disease | 24 (9.2) | 20 (11.5) | 1 (6.7) | 0 (0) | 0 (0) |
| Structural lung disease | 23 (8.8) | 12 (6.9) | 1 (6.7) | 2 (7.7) | 0 (0) |
| Congestive heart failure | 16 (6.1) | 10 (5.7) | 2 (13.3) | 1 (3.8) | 1 (8.3) |
| Myocardial infarction | 15 (5.7) | 10 (5.7) | 3 (20.0) | 1 (3.8) | 0 (0) |
| Chronic liver disease | 15 (5.7) | 7 (4.0) | 1 (6.7) | 3 (11.5) | 1 (8.3) |
| Cerebrovascular disease | 14 (5.4) | 9 (5.2) | 0 (0) | 1 (3.8) | 0 (0) |
| Rheumatic/connective tissue disease | 14 (5.4) | 8 (4.6) | 1 (6.7) | 1 (3.8) | 1 (8.3) |
| Gastric ulcer | 9 (3.4) | 7 (4.0) | 0 (0) | 1 (3.8) | 0 (0) |
| Hemiplegia or paraplegia | 9 (3.4 | 7 (4.0) | 1 (6.7) | 1 (3.8) | 0 (0) |
| Dementia | 7 (2.7) | 7 (4.0) | 0 (0) | 0 (0) | 0 (0) |
| Acquired immunodeficiency syndrome | 3 (1.1) | 1 (0.6) | 0 (0) | 0 (0) | 1 (8.3) |
| Metastatic solid tumour | 2 (0.8) | 2 (1.1) | 0 (0) | 0 (0) | 0 (0) |
| Transplant recipient | 54 (20.7) | 23 (13.2) | 6 (40.0) | 4 (15.4) | 4 (33.3) |
| Solid | 33 (12.6) | 17 (9.8) | 1 (6.7) | 4 (15.4) | 1 (8.3) |
| Haematopoietic | 21 (8.0) | 6 (3.4) | 5 (33.3) | 0 (0) | 3 (25.0) |

^a^*Pseudomonas* spp. include (*n*): *P. putida* (13); *P. fluorescens* (1); *P. nitroreducens* (1). ^b^Other Enterobacterales include (*n*): *Serratia marcescens* (5); *Enterobacter cloacae* (3); *Klebsiella oxytoca* (2); *Citrobacter freundii* (1); other *Serratia* spp. (1).

**Table S2.** Patients’ baseline characteristics and carbapenemase enzymes of baseline Gram-negative bacteria in the overall primary analysis population (*N*=261) and in patients with *P. aeruginosa* (*N*=174)

|  | Overall | *P. aeruginosa* |
| --- | --- | --- |
| Overall, *n* (%) | 261 (100) | 174 (66.7) |
| Admission type, *n* (%) |  |  |
| Emergency | 192 (73.6) | 131 (75.3) |
| Scheduled admission | 47 (18.0) | 24 (13.8) |
| Other | 22 (8.4) | 19 (10.9) |
| Ventilation for COVID-19-related symptoms, *n/N’* (%) | 53/63 (84.1) | 37/46 (80.4) |
| Hospital length of stay, days, median (IQR) | 69.0 (36–115) | 73.5 (40–121) |
| ICU length of stay, days, median (IQR) | 63.0 (33–91) | 64.0 (37–92) |
| Resistance mechanisms, *n* (%)^a^ |  |  |
| Not described | 129 (49.4) | 91 (52.3) |
| Class A β-lactamase | 4 (1.5) | 1 (0.6) |
| KPC | 2 (50.0) | 0 (0) |
| Other | 2 (50.0) | 1 (100) |
| Class B β-lactamase | 98 (37.5) | 73 (42.0) |
| IMP | 22 (22.4) | 20 (27.4) |
| NDM | 4 (4.1) | 0 (0) |
| Unknown MBL | 12 (12.2) | 10 (13.7) |
| VIM | 60 (61.2) | 43 (58.9) |
| Class C β-lactamase | 3 (1.1) | 3 (1.7) |
| Class D β-lactamase | 8 (3.1) | 1 (0.6) |
| Other | 1 (12.5) | 1 (100) |
| OXA-48-like | 7 (87.5) | 0 (0) |
| Double carbapenemase | 11 (4.2) | 2 (1.1) |
| L1/L2 β-lactamases | 3 (1.1) | 0 (0) |
| Unknown | 5 (1.9) | 3 (1.7) |

*N*’, total number of patients in the category.
COVID-19, coronavirus disease-2019; IMP, imipenemase metallo-β-lactamase; IQR, interquartile range; KPC, *Klebsiella pneumoniae* carbapenemase; MBL, metallo-β-lactamase; NDM, New Delhi metallo-β-lactamase; OXA, oxacillinase; VIM, Verona integron-encoded metallo-β-lactamase.
^a^Resistance mechanisms and presence of beta-lactamases were reported in the medical charts.

**Table S3.** Patients’ baseline demographics and clinical characteristics, resistance profile of baseline Gram-negative pathogens, pattern of cefiderocol use, and prior and concomitant antibiotic use by Gram-negative bacterial species in patients with *Pseudomonas* spp. (*N*=15), *K. pneumoniae* (*N*=26), and Other Enterobacterales (*N*=12)

| Overall *N*=261 | *Pseudomonas* spp.^a^ | *K. pneumoniae* | Other Enterobacterales^b^ |
| --- | --- | --- | --- |
| Patients, *n* (%) | 15 (5.7) | 26 (10.0) | 12 (4.6) |
| Age (years), median (IQR) | 59 (54.0–67.0) | 64.5 (49.0–70.0) | 51 (44.5–58.5) |
| Sex (male), *n* (%) | 11 (73.3) | 21 (80.8) | 9 (75.0) |
| Admission type, *n* (%) |  |  |  |
| Emergency | 8 (53.3) | 23 (88.5) | 8 (66.7) |
| Scheduled admission | 7 (46.7) | 2 (7.7) | 4 (33.3) |
| Other | 0 (0) | 1 (3.8) | 0 (0) |
| CCI score, median (IQR) | 4.00 (2.0–5.0) | 3.00 (2.0–4.0) | 2.50 (1.0–4.0) |
| SOFA score, median (IQR) | 9.0 (7.0–11.0) | 9.0 (6.0–12.0) | 9.0 (7.0–10.0) |
| APACHE II score, median (IQR) | 16 (8.0–24.0) | 13 (11.0–19.0) | 17 (12.0–24.0) |
| ICU, *n* (%) | 2 (13.3) | 18 (69.2) | 10 (83.3) |
| Mechanical ventilation at baseline, *n* (%) | 2 (13.3) | 16 (61.5) | 5 (41.7) |
| Symptomatic COVID-19 during hospitalisation, *n* (%) | 1 (6.7) | 8 (30.8) | 3 (25.0) |
| Ventilation for COVID-19-related symptoms, *n/N’* (%) | 1/1 (100) | 8/8 (100) | 2/3 (66.7) |
| Hospital length of stay, days, median (IQR) | 34.0 (27–51) | 58 (31–98) | 70.5 (39–112.5) |
| ICU length of stay, days, median (IQR) | 34.5 (32–37) | 49.5 (27–64) | 45.5 (22–75) |
| Septic shock, *n* (%) | 1 (6.7) | 13 (50.0) | 3 (25.0) |
| ECMO, *n* (%) | 0 (0) | 1 (3.8) | 0 (0) |
| RRT, *n/N* (%) | 0/15 (0) | 10/26 (38.5) | 5/12 (41.7) |
| Creatinine clearance <60 mL/min, *n/N’* (%)^c^ | 4/15 (26.7) | 5/13 (38.5) | 1/7 (14.3) |
| Immunosuppressed, *n* (%)^d^ | 9 (60.0) | 5 (19.2) | 4 (33.3) |
| Primary infection site, *n* (%) |  |  |  |
| Respiratory | 2 (13.3) | 16 (61.5) | 5 (41.7) |
| Urinary | 6 (40.0) | 0 (0) | 1 (8.3) |
| Intra-abdominal | 3 (20.0) | 7 (26.9) | 2 (16.7) |
| Skin and soft tissue | 3 (20.0) | 0 (0) | 1 (8.3) |
| Bloodstream | 1 (6.7) | 3 (11.5) | 3 (25.0) |
| Bloodstream (catheter related) | 1 (6.7) | 0 (0) | 2 (16.7) |
| Bloodstream (unknown source) | 0 (0) | 3 (11.5) | 1 (8.3) |
| Bone and joint | 0 (0) | 0 (0) | 0 (0) |
| Other | 0 (0) | 0 (0) | 0 (0) |
| Secondary bloodstream infection, *n* (%) | 1 (7.1) | 5 (21.7) | 4 (44.4) |
| Polymicrobial infection, *n* (%)^e^ | 1 (6.7) | 8 (30.8) | 2 (16.7) |
| Previous colonisation, *n/N’* (%) | 5/15 (33.3) | 19/25 (76.0) | 6/12 (50.0) |
| Meropenem resistant, *n/N’* (%)^f^ | 14/14 (100) | 14/20 (70.0) | 7/10 (70.0) |
| Ceftazidime-avibactam resistant, *n/N’* (%)^g^ | 9/10 (90.0) | 9/18 (50.0) | 9/9 (100) |
| Ceftolozane-tazobactam resistant, *n/N’* (%)^g^ | 6/7 (85.7) | 7/8 (87.5) | 5/5 (100) |
| Resistant to ceftazidime-avibactam and ceftolozane-tazobactam, *n/N’* (%)^h^ | 6/6 (100) | 7/8 (87.5) | 5/5 (100) |
| Prior antibiotics, *n* (%)^i^ | 12 (80.0) | 24 (92.3) | 6 (50.0) |
| Number of prior courses of antibiotic treatments, median (IQR) | 1.0 (1.0–3.0) | 3.0 (2.0–3.0) | 2.0 (2.0–2.8) |
| 1, *n* (%) | 8 (66.7) | 5 (23.8) | 0 (0) |
| 2, *n* (%) | 0 (0) | 5 (23.8) | 4 (66.7) |
| ≥3, *n* (%) | 4 (33.3) | 11 (52.4) | 2 (33.3) |
| None, *n* (%) | 3 (20.0) | 2 (7.7) | 6 (50.0) |
| Unknown, *n* | 0 | 3 | 0 |
| Duration of prior antibiotic treatment (days), median (IQR) | 5.9 (3.9–8.5) | 8 (5.0–11.4) | 5.4 (3.5–8.2) |
| ≤3, *n/N’* (%) | 3/12 (25.0) | 2/21 (9.5) | 1/6 (16.7) |
| 4–7, *n/N’* (%) | 6/12 (50.0) | 7/21 (33.3) | 3/6 (50.0) |
| >7, *n/N’* (%) | 3/12 (25.0) | 12/21 (57.1) | 2/6 (33.3) |
| Rationale for administration of cefiderocol, *n* (%)^j^ |  |  |  |
| Resistance to all tested antibiotics | 12 (80.0) | 13 (50.0) | 7 (58.3) |
| Treatment failure of prior antibiotics | 5 (33.3) | 18 (69.2) | 1 (8.3) |
| Adverse events to other susceptible antibiotics | 0 (0) | 2 (7.7) | 1 (8.3) |
| Other | 0 (0) | 1 (3.8) | 3 (25.0) |
| Cefiderocol as first-line therapy, *n* (%) | 3 (20.0) | 2 (7.7) | 6 (50.0) |
| Duration of cefiderocol treatment (days), median (IQR) | 10.0 (5.0–14.0) | 10.0 (6.0–14.0) | 10.0 (6.5–14.0) |
| Combination therapy given with cefiderocol, *n* (%)^k^ | 2 (13.3) | 10 (38.5) | 5 (41.7) |
| Number of antibiotics concomitantly with cefiderocol, *n* (%) |  |  |  |
| 1 | 1 (6.7) | 5 (19.2) | 1 (8.3) |
| 2 | 1 (6.7) | 2 (7.7) | 3 (25.0) |
| ≥3 | 0 | 3 (11.5) | 1 (8.3) |
| Cefiderocol dosing, *n* (%) |  |  |  |
| Every 4 hours | 0 (0.0%) | 0 (0.0%) | 1 (8.3%) |
| Every 6 hours | 2 (13.3%) | 3 (11.5%) | 0 (0.0%) |
| Every 8 hours | 12 (80.0%) | 20 (76.9%) | 10 (83.3%) |
| Every 12 hours | 1 (6.7%) | 3 (11.5%) | 1 (8.3%) |
| Other | 0 (0.0%) | 0 (0.0%) | 0 (0.0%) |

*N*’, total number of patients in the category.
APACHE, acute physiology and chronic health evaluation; CCI, Charlson Comorbidity Index; COVID-19, coronavirus disease-2019; ECMO, extracorporeal membrane oxygenation; ICU, intensive care unit; IQR, interquartile range; RRT, renal replacement therapy; SOFA, sequential organ failure assessment.
^a^*Pseudomonas* spp. include (*n*): *P. putida* (13); *P. fluorescens* (1); *P. nitroreducens* (1). ^b^Other Enterobacterales include (*n*): *Serratia marcescens* (5); *Enterobacter cloacae* (3); *Klebsiella oxytoca* (2); *Citrobacter freundii* (1); other *Serratia* spp. (1). ^c^Excluding patients on RRT; denominator excludes missing data. ^d^Transplant recipient, immunosuppressive treatment (e.g. high-dose corticosteroids, calcineurin inhibitors, anti-CD20, IL-1 inhibitors and IL-6 inhibitors).
^e^Primary pathogen in polymicrobial infections, for which cefiderocol was requested, was confirmed by the treating physician.
^f^Susceptibility test results were reported at local site.
^g^Includes patients with confirmed (non-missing) susceptibility result; susceptibility was reported in the medical charts.
^h^Includes patients with susceptibility test results for both ceftazidime-avibactam and ceftolozane-tazobactam.
^i^Prior antibiotics were given to a total of 219 patients; data are shown for 212 patients with a full data set information was missing for 7 patients.
^j^Not mutually exclusive; physicians could select ≥1 option.
^k^Includes antibiotics with Gram-negative coverage that have been started before, concomitantly or during the same treatment period.

**Table S4**. Patients’ baseline demographic and clinical characteristics, resistance profile of baseline Gram-negative pathogens, prior antibiotic treatment and cefiderocol treatment by infection site in the overall primary analysis population (*N*=261)

|  | Respiratory | IAI | UTI | SSTI | BSI | Bone and joint infection | Other |
| --- | --- | --- | --- | --- | --- | --- | --- |
|  | *N*=125 | *N*=38 | *N*=38 | *N*=26 | *N*=24 | *N*=6 | *N*=4 |
| Age (years), median (IQR) | 60 (46–66) | 62 (51–71) | 66.5 (55–75) | 61.5 (55–68) | 57.5 (45–64.5) | 62 (60–64) | 54.5 (42–63.5) |
| Sex (male), *n* (%) | 97 (77.6) | 31 (81.6) | 26 (68.4) | 19 (73.1) | 21 (87.5) | 6 (100) | 2 (50.0) |
| Admission type, *n* (%) |  |  |  |  |  |  |  |
| Emergency | 103 (82.4) | 23 (60.5) | 32 (84.2) | 19 (73.1) | 13 (54.2) | 1 (16.7) | 1 (25.0) |
| Scheduled admission | 10 (8.0) | 14 (36.8) | 5 (13.2) | 3 (11.5) | 9 (37.5) | 3 (50.0) | 3 (75.0) |
| Other | 12 (9.6) | 1 (2.6) | 1 (2.6) | 4 (15.4) | 2 (8.3) | 2 (33.3) | 0 (0) |
| Any pre-existing medical condition, *n* (%) | 82 (65.6) | 33 (86.8) | 32 (84.2) | 20 (76.9) | 23 (95.8) | 5 (83.3) | 4 (100) |
| Diabetes mellitus | 24 (19.2) | 5 (13.2) | 9 (23.7) | 10 (38.5) | 7 (29.2) | 2 (33.3) | 1 (25.0) |
| Solid/haematological tumour | 13 (10.4) | 18 (47.4) | 13 (34.2) | 4 (15.4) | 11 (45.8) | 1 (16.7) | 2 (50.0) |
| Chronic renal disease | 12 (9.6) | 6 (15.8) | 7 (18.4) | 4 (15.4) | 3 (12.5) | 2 (33.3) | 0 (0) |
| COPD | 14 (11.2) | 3 (7.9) | 4 (10.5) | 4 (15.4) | 2 (8.3) | 0 (0) | 0 (0) |
| Chronic liver disease | 6 (4.8) | 6 (15.8) | 1 (2.6) | 1 (3.8) | 1 (4.2) | 0 (0) | 0 (0) |
| Structural pulmonary disease | 20 (16.0) | 0 (0) | 0 (0) | 3 (11.5) | 0 (0) | 0 (0) | 0 (0) |
| Peripheral vascular disease | 7 (5.6) | 3 (7.9) | 3 (7.9) | 6 (23.1) | 4 (16.7) | 1 (16.7) | 0 (0) |
| CCI score, median (IQR) | 2.0 (1.0–4.00) | 4.0 (2.0–5.0) | 4.0 (3.0–5.0) | 3.0 (2.0–5.0) | 3.0 (2.0–6.0) | 4.0 (3.0–4.0) | 3.0 (2.5–3.0) |
| SOFA score at baseline, median (IQR) | 7.0 (4.0–11.0)  N’=82 | 10.0 (6.0–13.0)  N’=11 | 5.5 (4.0–6.0)  N’=6 | 11.0 (4.0–12.0)  N’=6 | 8.0 (7.0-10.0)  N’=11 | 12.0 (12.0–12.0)  N’=1 | 8.0 (8.0–8.0)  N’13 |
| APACHE II score on admission to ICU,  median (IQR) | 15 (10–21)  N’=101 | 12.5 (8.5–17.5)  N’=16 | 20 (12–30.5)  N’=8 | 20.5 (15.5–24)  N’=8 | 17.0 (15-23)  N’=15 | 20 (20–20)  N’=1 | 2 (0–23)  N’=3 |
| ICU, *n* (%) | 112 (89.6) | 17 (44.7) | 7 (18.4) | 9 (34.6) | 16 (66.7) | 1 (16.7) | 3 (75.0) |
| Mechanical ventilation at baseline, *n* (%) | 89 (71.2) | 12 (31.6) | 5 (13.2) | 4 (15.4) | 9 (37.5) | 1 (16.7) | 3 (75.0) |
| Symptomatic COVID-19 during hospitalisation, *n* (%) | 50 (40.0) | 3 (7.9) | 3 (7.9) | 3 (11.5) | 3 (12.5) | 0 (0) | 1 (25.0) |
| Ventilation for COVID-19-related symptoms, *n/N’* (%) | 48/50 (96.0) | 0/3 (0) | 1/3 (33.3) | 2/3 (66.7) | 2/3 (66.7) | 0/0 (0) | 0/1 (0) |
| Septic shock, *n* (%) | 45 (36.0) | 10 (26.3) | 1 (2.6) | 7 (26.9) | 8 (33.3) | 1 (16.7) | 1 (25.0) |
| ECMO, *n* (%) | 10 (8.0) | 0 (0) | 1 (2.6) | 1 (3.8) | 0 (0) | 0 (0) | 0 (0) |
| RRT, *n* (%) | 45 (36.0) | 9 (23.7) | 3 (7.9) | 5 (19.2) | 9 (37.5) | 0 (0) | 0 (0) |
| Creatinine clearance <60 mL/min, *n/N’* (%)^a^ | 16/78 (20.5) | 8/25 (32.0) | 15/29 (51.7) | 7/21 (33.3) | 4/15 (26.7) | 3/5 (60.0) | 1/4 (25.0) |
| Immunosuppressed, *n* (%)^b^ | 28 (22.4) | 15 (39.5) | 16 (42.1) | 6 (23.1) | 13 (54.2) | 0 (0) | 1 (25.0) |
| Secondary bloodstream infection, *n* (%) | 26 (20.8) | 7 (18.4) | 4 (10.5) | 7 (26.9) | 0 (0) | 1 (16.7) | 0 (0) |
| Polymicrobial infection, *n* (%)^c^ | 30 (24.0) | 8 (21.1) | 5 (13.2) | 3 (11.5) | 4 (16.7) | 0 (0) | 1 (25.0) |
| Previous colonisation, *n/N’* (%) | 75/123 (61.0) | 20/38 (52.6) | 11/38 (28.9) | 13/25 (52.0) | 12/21 (57.1) | 3/6 (50.0) | 1/4 (25.0) |
| Baseline Gram-negative species, *n* (%) |  |  |  |  |  |  |  |
| *Pseudomonas aeruginosa* | 81 (64.8) | 23 (60.5) | 28 (73.7) | 22 (84.6) | 11 (45.8) | 6 (100) | 3 (75.0) |
| *Pseudomonas* spp. | 2 (1.6) | 3 (7.9) | 6 (15.8) | 3 (11.5) | 1 (4.2) | 0 (0) | 0 (0) |
| *Klebsiella pneumoniae* | 16 (12.8) | 7 (18.4) | 0 (0) | 0 (0) | 3 (12.5) | 0 (0) | 0 (0) |
| Other Enterobacterales | 5 (4.0) | 2 (5.3) | 1 (2.6) | 1 (3.8) | 3 (12.5) | 0 (0) | 0 (0) |
| *Stenotrophomonas maltophilia* | 11 (8.8) | 2 (5.3) | 1 (2.6) | 0 (0) | 5 (20.8) | 0 (0) | 1 (25.0) |
| Other non-fermenting species |  |  |  |  |  |  |  |
| *Achromobacter* spp. | 2 (1.6) | 0 (0) | 2 (5.3) | 0 (0) | 1 (4.2) | 0 (0) | 0 (0) |
| *Burkholderia cepacia* complex | 7 (5.6) | 1 (2.6) | 0 (0) | 0 (0) | 0 (0) | 0 (0) | 0 (0) |
| *Ralstonia mannitolilytica* | 1 (0.8) | 0 (0) | 0 (0) | 0 (0) | 0 (0) | 0 (0) | 0 (0) |
| Meropenem resistant, *n/N’* (%)^d^ | 93/103 (90.3) | 30/31 (96.8) | 26/27 (96.3) | 22/24 (91.7) | 10/13 (76.9) | 4/4 (100) | 4/4 (100) |
| Ceftazidime-avibactam resistant, *n/N’* (%)^e^ | 65/77 (84.4) | 22/29 (75.9) | 15/19 (78.9) | 15/17 (88.2) | 13/15 (86.7) | 3/3 (100) | 1/1 (100) |
| Ceftolozane-tazobactam resistant, *n/N’* (%)^e^ | 45/65 (69.2) | 14/19 (73.7) | 13/14 (92.9) | 16/18 (88.9) | 9/12 (75.0) | 1/3 (33.3) | 1/1 (100) |
| Resistant to ceftazidime-avibactam and ceftolozane-tazobactam, *n/N’* (%)^f^ | 46/64 (71.9) | 13/17 (76.5) | 13/14 (92.9) | 16/18 (88.9) | 9/12 (75.0) | 1/3 (33.3) | 1/1 (100) |
| Prior antibiotics^g^ | *N*’=100 | *N*’=34 | *N*’=33 | *N*’=19 | *N*’=16 | *N*’=6 | *N*’=4 |
| Number of prior courses of antibiotic treatments, median (IQR) | 3 (2.0–4.0) | 2 (2.0–3.0) | 2 (1.0–3.0) | 2 (1.0–4.0) | 2.0 (1.5–3.5) | 2 (2.0–3.0) | 3 (2.0–5.0) |
| 1, *n* (%) | 20 (20.0) | 8 (23.5) | 15 (45.5) | 6 (31.6) | 4 (25.0) | 1 (16.7) | 0 (0) |
| 2, *n* (%) | 27 (27.0) | 11 (32.3) | 9 (27.3) | 5 (26.3) | 5 (31.3) | 3 (50.0) | 2 (50.0) |
| ≥3, *n* (%) | 53 (53.0) | 15 (44.1) | 9 (27.3) | 8 (42.1) | 7 (43.8) | 2 (33.3) | 2 (50.0) |
| None, *n* (%) | 21 (16.8) | 3 (7.9) | 5 (13.2) | 6 (23.1) | 7 (29.2) | 0 (0) | 0 (0) |
| Unknown, *n* | 4 | 1 | 0 | 1 | 1 | 0 | 0 |
| Duration of prior antibiotic treatment (days), median IQR) | 6.7 (4.0–10.4) | 9 (3.3–12.0) | 4 (3.0–7.0) | 5 (4.0–11.0) | 4.7 (2.4–7.9) | 4.8 (1.0–17.0) | 7.9 (3.3–15.4) |
| ≤3, *n* (%) | 20 (20.0) | 8 (23.5) | 13 (39.4) | 4 (21.1) | 7 (43.8) | 2 (33.3) | 1 (25.0) |
| 4–7, *n* (%) | 37 (37.0) | 6 (17.6) | 12 (36.4) | 8 (42.1) | 4 (25.0) | 2 (33.3) | 1 (25.0) |
| >7, *n* (%) | 43 (43.0) | 20 (58.8) | 8 (24.2) | 7 (36.8) | 5 (31.3) | 2 (33.3) | 2 (50.0) |
| Rationale for administration of cefiderocol, *n* (%)^h^ |  |  |  |  |  |  |  |
| Resistance to all tested antibiotics | 85 (68.0) | 24 (63.2) | 22 (57.9) | 17 (65.4) | 16 (66.7) | 3 (50.0) | 2 (50.0) |
| Treatment failure of prior antibiotics | 59 (47.2) | 19 (50.0) | 18 (47.4) | 8 (30.8) | 9 (37.5) | 1 (16.7) | 2 (50.0) |
| Adverse events to other susceptible antibiotics | 7 (5.6) | 4 (10.5) | 3 (7.9) | 4 (15.4) | 1 (4.2) | 1 (16.7) | 1 (25.0) |
| Other | 13 (10.4) | 2 (5.3) | 2 (5.3) | 3 (11.5) | 3 (12.5) | 2 (33.3) | 1 (25.0) |
| Cefiderocol as first-line therapy, *n* (%) | 21 (16.8) | 3 (7.9) | 5 (13.2) | 6 (23.1) | 7 (29.2) | 0 (0) | 0 (0) |
| Duration of cefiderocol treatment (days), median (IQR) | 10.0 (7.0–14.0) | 10.0 (7.0–17.0) | 8.0 (7.0–13.0) | 12.5 (9.0–14.0) | 11.5 (5.0–15.0) | 16.5 (8.0–17.0) | 18.0 (9.5–24.5) |
| Combination therapy given with cefiderocol, *n* (%)^i^ | 58 (46.4) | 14 (36.8) | 7 (18.4) | 4 (15.4) | 4 (16.7) | 2 (33.3) | 2 (50.0) |
| Number of antibiotics concomitantly with cefiderocol, *n* (%) |  |  |  |  |  |  |  |
| 1 | 26 (20.8) | 5 (13.2) | 4 (10.5) | 3 (11.5) | 0 (0) | 2 (33.3) | 1 (25.0) |
| 2 | 12 (9.6) | 6 (15.8) | 2 (5.3) | 1 (3.8) | 3 (12.5) | 0 (0) | 1 (25.0) |
| ≥3 | 20 (16.0) | 3 (7.9) | 1 (2.6) | 0 (0) | 1 (4.2) | 0 (0) | 0 (0) |
| Cefiderocol dosing, *n* (%) |  |  |  |  |  |  |  |
| Every 4 hours | 2 (1.6%) | 0 (0.0%) | 1 (2.6%) | 0 (0.0%) | 0 (0.0%) | 0 (0.0%) | 0 (0.0%) |
| Every 6 hours | 14 (11.2%) | 2 (5.3%) | 4 (10.5%) | 2 (7.7%) | 1 (4.2%) | 1 (16.7%) | 1 (25.0%) |
| Every 8 hours | 99 (79.2%) | 33 (86.8%) | 29 (76.3%) | 22 (84.6%) | 22 (91.7%) | 5 (83.3%) | 3 (75.0%) |
| Every 12 hours | 10 (8.0%) | 3 (7.9%) | 4 (10.5%) | 2 (7.7%) | 1 (4.2%) | 0 (0.0%) | 0 (0.0%) |
| Other | 0 (0.0%) | 0 (0.0%) | 0 (0.0%) | 0 (0.0%) | 0 (0.0%) | 0 (0.0%) | 0 (0.0%) |

*N*’, total number of patients in the category.
APACHE, acute physiology and chronic health evaluation; BSI, bloodstream infection; CCI, Charlson Comorbidity Index; COPD, chronic obstructive pulmonary disease; COVID-19, coronavirus disease-2019; ECMO, extracorporeal membrane oxygenation; IAI, intra-abdominal infection; ICU, intensive care unit; IQR, interquartile range; RRT, renal replacement therapy; SOFA, sequential organ failure assessment; SSTI, skin and soft tissue infection; UTI, urinary tract infection.
^a^Excluding patients on RRT; denominator excludes missing data. ^b^Transplant recipient, immunosuppressive treatment (e.g. high-dose corticosteroids, calcineurin inhibitors, anti-CD20, IL-1 inhibitors and IL-6 inhibitors).
^c^Primary pathogen in polymicrobial infections, for which cefiderocol was requested, was confirmed by the treating physician.
^d^Susceptibility test results were reported at local site.
^e^Includes patients with confirmed (non-missing) susceptibility result; susceptibility was reported in the medical charts.
^f^Includes patients with susceptibility test results for both ceftazidime-avibactam and ceftolozane-tazobactam.
^g^Prior antibiotics were given to a total of 219 patients; data are shown for 212 patients with a full data set; information is missing for 7 patients. List of prior antibiotics is included in **Table S5**.
^h^Not mutually exclusive; physicians could select ≥1 option.
^i^Includes antibiotics with Gram-negative coverage that have been started before, concomitantly or during the same treatment period.

**Table S5.** Antibiotics with Gram-negative activity given prior to cefiderocol treatment in the overall primary analysis population (*N*=261) and in patients with *P. aeruginosa* (*N*=174), *Pseudomonas* spp. (*N*=15), *K. pneumoniae* (*N*=26), and Other Enterobacterales (*N*=12)

| Prior antibiotics in the primary analysis population, *n* (%) | Overall | *P. aeruginosa* | *Pseudomonas* spp.^a^ | *K. pneumoniae* | Other Enterobacterales^b^ |
| --- | --- | --- | --- | --- | --- |
|  | *N*=261 | *N*=174 | *N*=15 | *N*=26 | *N*=12 |
| Colistin | 95 (36.4) | 81 (46.6) | 1 (6.7) | 7 (26.9) | 1 (8.3) |
| Meropenem | 80 (30.7) | 55 (31.6) | 5 (33.3) | 7 (26.9) | 3 (25.0) |
| Ceftazidime-avibactam | 81 (31.0) | 55 (31.6) | 1 (6.7) | 13 (50.0) | 4 (33.3) |
| Amikacin | 46 (17.6) | 35 (20.1) | 2 (13.3) | 5 (19.2) | 2 (16.7) |
| Aztreonam | 43 (16.5) | 36 (20.7) | 0 (0) | 2 (7.7) | 1 (8.3) |
| Piperacillin-tazobactam | 37 (14.2) | 30 (17.2) | 2 (13.3) | 1 (3.8) | 0 (0) |
| Tigecycline | 22 (8.4) | 5 (2.9) | 1 (6.7) | 7 (26.9) | 3 (25.0) |
| Trimethoprim- sulfamethoxazole | 20 (7.7) | 5 (2.9) | 0 (0) | 3 (11.5) | 0 (0) |
| Ciprofloxacin | 18 (6.9) | 13 (7.5) | 0 (0) | 2 (7.7) | 0 (0) |
| Ceftazidime | 16 (6.1) | 10 (5.7) | 2 (13.3) | 0 (0) | 1 (8.3) |
| Ceftolozane-tazobactam | 12 (4.6) | 9 (5.2) | 1 (6.7) | 1 (3.8) | 0 (0) |
| Fosfomycin | 7 (2.7) | 3 (1.7) | 0 (0) | 3 (11.5) | 0 (0) |
| Amoxicillin-clavulanic acid | 6 (2.3) | 6 (3.4) | 0 (0) | 0 (0) | 0 (0) |
| Imipenem | 6 (2.3) | 6 (3.4) | 0 (0) | 0 (0) | 0 (0) |
| Cefotaxime | 4 (1.5) | 3 (1.7) | 0 (0) | 0 (0) | 1 (8.3) |
| Gentamicin | 2 (0.8) | 2 (1.1) | 0 (0) | 0 (0) | 0 (0) |
| Imipenem-relebactam | 1 (0.4) | 1 (0.6) | 0 (0) | 0 (0) | 0 (0) |
| Meropenem-vaborbactam | 1 (0.4) | 0 (0.0) | 0 (0) | 1 (3.8) | 0 (0) |
| Other | 57 (21.8) | 39 (22.4) | 5 (33.3) | 4 (15.4) | 0 (0) |

^a^*Pseudomonas* spp. include (*n*): *P. putida* (13); *P. fluorescens* (1); *P. nitroreducens* (1). ^b^Other Enterobacterales include (*n*): *Serratia marcescens* (5); *Enterobacter cloacae* (3); *Klebsiella oxytoca* (2); *Citrobacter freundii* (1); other *Serratia* spp. (1).

**Table S6.** Concomitant antibiotics with Gram-negative activity given during cefiderocol treatment in the overall primary analysis population (*N*=261) and in patients with *P. aeruginosa* (*N*=174), *Pseudomonas* spp. (*N*=15), *K. pneumoniae* (*N*=26), and Other Enterobacterales (*N*=12)

| Concomitant antibiotics^a^ in the primary analysis population, *n* (%) | Overall | *P. aeruginosa* | *Pseudomonas* spp.^b^ | *K. pneumoniae* | Other Enterobacterales^c^ |
| --- | --- | --- | --- | --- | --- |
|  | *N*=261 | *N*=174 | *N*=15 | *N*=26 | *N*=12 |
| Colistin | 51 (19.5) | 40 (23.0) | 0 (0.0) | 6 (23.1) | 1 (8.3) |
| Ceftazidime-avibactam | 19 (7.3) | 13 (7.5) | 0 (0.0) | 1 (3.8) | 2 (16.7) |
| Amikacin | 17 (6.5) | 11 (6.3) | 1 (6.7) | 2 (7.7) | 2 (16.7) |
| Meropenem | 16 (6.1) | 6 (3.4) | 1 (6.7) | 2 (7.7) | 2 (16.7) |
| Tigecycline | 14 (5.4) | 1 (0.6) | 1 (6.7) | 4 (15.4) | 3 (25.0) |
| Ciprofloxacin | 7 (2.7) | 4 (2.3) | 0 (0.0) | 1 (3.8) | 0 (0.0) |
| Aztreonam | 8 (3.1) | 6 (3.4) | 0 (0.0) | 0 (0.0) | 1 (8.3) |
| Trimethoprim-sulfamethoxazole | 9 (3.4) | 2 (1.1) | 0 (0.0) | 0 (0.0) | 0 (0.0) |
| Piperacillin-tazobactam | 7 (2.7) | 5 (2.9) | 0 (0.0) | 0 (0.0) | 0 (0.0) |
| Ceftazidime | 6 (2.3) | 3 (1.7) | 0 (0.0) | 0 (0.0) | 1 (8.3) |
| Fosfomycin | 4 (1.5) | 1 (0.6) | 0 (0.0) | 3 (11.5) | 0 (0.0) |
| Ceftolozane-tazobactam | 3 (1.1) | 2 (1.1) | 0 (0.0) | 0 (0.0) | 0 (0.0) |
| Imipenem | 1 (0.4) | 1 (0.6) | 0 (0.0) | 0 (0.0) | 0 (0.0) |
| Other | 21 (8.0) | 14 (8.0) | 0 (0.0) | 1 (3.8) | 0 (0.0) |

^a^Concomitant antibiotics include agents with activity against Gram-negative bacteria, which were initiated prior to cefiderocol and continued during cefiderocol treatment or were initiated simultaneously or were administered following initiation of cefiderocol treatment.
^b^*Pseudomonas* spp. include (*n*): *P. putida* (13); *P. fluorescens* (1); *P. nitroreducens* (1). ^c^Other Enterobacterales include (*n*): *Serratia marcescens* (5); *Enterobacter cloacae* (3); *Klebsiella oxytoca* (2); *Citrobacter freundii* (1); other *Serratia* spp. (1).

**Table S7.** Clinical cure, all-cause mortality, and clinical success rates, in the overall primary analysis population (*N*=261) and in patients with *Pseudomonas aeruginosa* (*N*=174), by baseline clinical characteristics, infection type, prior antibiotic treatment, antibiotic resistance status and cefiderocol use

| Baseline characteristics | Overall | Clinical cure at EOT | All-cause mortality at Day 28 | Composite clinical success |
| --- | --- | --- | --- | --- |
| Overall, *n/N* (%) | 261 (100) | 210 (80.5) | 56 (21.5) | 220 (84.3) |
| Immunosuppression^a^, *n/N’* (%) |  |  |  |  |
| No | 182 (69.7) | 149 (81.9) | 38 (20.9) | 156 (85.7) |
| Yes | 79 (30.3) | 61 (77.2) | 18 (22.8) | 64 (81.0) |
| Symptomatic COVID-19 during hospitalisation, *n/N’* (%) |  |  |  |  |
| No | 198 (75.9) | 161 (81.3) | 43 (21.7) | 166 (83.8) |
| Yes | 63 (24.1) | 49 (77.8) | 13 (20.6) | 54 (85.7) |
| Septic shock, *n/N’* (%) |  |  |  |  |
| No | 188 (72.0) | 160 (85.1) | 30 (16.0) | 166 (88.3) |
| Yes | 73 (28.0) | 50 (68.5) | 26 (35.6) | 54 (74.0) |
| ICU at the time of infection, *n/N’* (%) |  |  |  |  |
| No | 96 (36.8) | 88 (91.7) | 7 (7.3) | 93 (96.9) |
| Yes | 165 (63.2) | 122 (73.9) | 49 (29.7) | 127 (77.0) |
| Mechanical ventilation baseline, *n/N’* (%) |  |  |  |  |
| No | 138 (52.9) | 123 (89.1) | 15 (10.9) | 129 (93.5) |
| Yes | 123 (47.1) | 87 (70.7) | 41 (33.3) | 91 (74.0) |
| RRT, *n/N’* (%) |  |  |  |  |
| No | 190 (72.8) | 161 (84.7) | 29 (15.3) | 167 (87.9) |
| Yes | 71 (27.2) | 49 (69.0) | 27 (38.0) | 53 (74.6) |
| Polymicrobial infection, *n/N’* (%) |  |  |  |  |
| No | 210 (80.5) | 171 (81.4) | 44 (21.0) | 178 (84.8) |
| Yes | 51 (19.5) | 39 (76.5) | 12 (23.5) | 42 (82.4) |
| Previous colonisation, *n/N’* (%) |  |  |  |  |
| No | 120 (47.1) | 96 (80.0) | 24 (20.0) | 100 (83.3) |
| Yes | 135 (52.9) | 110 (81.5) | 31 (23.0) | 115 (85.2) |
| Missing data, (*n*) | 6 | 4 | 2 | 5 |
| Secondary bloodstream infection, *n/N’* (%)^b^ |  |  |  |  |
| No | 188 (72.0) | 154 (81.9) | 39 (20.7) | 159 (84.6) |
| Yes | 45 (17.2) | 34 (75.6) | 11 (24.4) | 38 (84.4) |
| Not assessed | 5 (1.9) | 4 (80.0) | 1 (20.0) | 4 (80.0) |
| Number of prior courses of antibiotic treatments, *n/N’* (%) | *N*’=212 | *N*’=168 | *N*’=47 | *N*’=178 |
| 1 | 54 (25.5) | 47 (87.0) | 9 (16.7) | 48 (88.9) |
| 2 | 62 (29.2) | 52 (83.9) | 10 (16.1) | 54 (87.1) |
| ≥3 | 96 (45.3) | 69 (71.9) | 28 (29.2) | 76 (79.2) |
| Prior antibiotic treatment, *n/N’* (%) |  |  |  |  |
| Colistin | 95 (100) | 74 (77.9) | 25 (26.3) | 78 (82.1) |
| Meropenem | 80 (100) | 58 (72.5) | 23 (28.8) | 63 (78.8) |
| Ceftazidime-avibactam | 81 (100) | 59 (72.8) | 23 (28.4) | 62 (76.5) |
| Ceftolozane-tazobactam | 12 (100) | 9 (75.0) | 2 (16.7) | 10 (83.3) |
| *Pseudomonas aeruginosa, n/N (%)* | 174 (100) | 147 (84.5) | 30 (17.2) | 155 (89.1) |
| Immunosuppression^a^, *n/N’* (%) |  |  |  |  |
| No | 133 (76.4) | 113 (85.0) | 21 (15.8) | 120 (90.2) |
| Yes | 41 (23.6) | 34 (82.9) | 9 (22.0) | 35 (85.4) |
| Symptomatic COVID-19 during hospitalisation, *n/N’* (%) |  |  |  |  |
| No | 128 (73.6) | 111 (86.7) | 22 (17.2) | 115 (89.8) |
| Yes | 46 (26.4) | 36 (78.3) | 8 (17.4) | 40 (87.0) |
| Septic shock, *n/N’* (%) |  |  |  |  |
| No | 127 (73.0) | 115 (90.6) | 15 (11.8) | 119 (93.7) |
| Yes | 47 (27.0) | 32 (68.1) | 15 (31.9) | 36 (76.6) |
| ICU at the time of infection, *n/N’* (%) |  |  |  |  |
| No | 65 (37.4) | 62 (95.4) | 4 (6.2) | 65 (100) |
| Yes | 109 (62.6) | 85 (78.0) | 26 (23.9) | 90 (82.6) |
| Mechanical ventilation at the time of infection, *n/N’* (%) |  |  |  |  |
| No | 95 (54.6) | 87 (91.6) | 10 (10.5) | 91 (95.8) |
| Yes | 79 (45.4) | 60 (75.9) | 20 (25.3) | 64 (81.0) |
| Polymicrobial infection, *n/N’* (%) |  |  |  |  |
| No | 138 (79.3) | 119 (86.2) | 23 (16.7) | 124 (89.9) |
| Yes | 36 (20.7) | 28 (77.8) | 7 (19.4) | 31 (86.1) |
| Previous colonisation, *n/N’* (%) |  |  |  |  |
| No | 82 (48.0) | 68 (82.9) | 13 (15.9) | 72 (87.8) |
| Yes | 89 (52.0) | 77 (86.5) | 17 (19.1) | 80 (89.9) |
| Missing data, (*n*) | 3 | 2 | 1 | 3 |
| Secondary bloodstream infection, *n/N’* (%)^c^ |  |  |  |  |
| No | 132 (75.9) | 114 (86.4) | 23 (17.4) | 118 (89.4) |
| Yes | 28 (16.1) | 20 (71.4) | 5 (17.9) | 24 (85.7) |
| Not assessed | 4 (2.3) | 3 (75.0) | 1 (25.0) | 3 (75.0) |
| Meropenem resistance, *n/N’* (%) |  |  |  |  |
| Yes | 139 (79.9) | 119 (85.6) | 23 (16.5) | 125 (89.9) |
| Ceftolozane-tazobactam resistance, *n/N’* (%) |  |  |  |  |
| Yes | 75 (43.1) | 65 (86.7) | 10 (13.3) | 67 (89.3) |
| Ceftazidime-avibactam resistance, *n/N’* (%) |  |  |  |  |
| Yes | 96 (55.2) | 80 (83.3) | 18 (18.8) | 83 (86.5) |
| Cross-resistance to ceftazidime-avibactam and ceftolozane-tazobactam, *n/N’* (%) |  |  |  |  |
| Yes | 99 (56.9) | 82 (82.8) | 17 (17.2) | 85 (85.9) |
| Colistin resistance, *n/N’* (%) |  |  |  |  |
| Yes | 16 (9.2) | 13 (81.3) | 3 (18.8) | 13 (81.3) |
| Number of prior courses of antibiotic treatments, *n/N’* (%) | *N*=147 | *N*=122 | *N*=27 | *N*=130 |
| 1 | 37 (25.2) | 35 (94.6) | 5 (13.5) | 35 (94.6) |
| 2 | 43 (29.3) | 36 (83.7) | 8 (18.6) | 37 (86.0) |
| ≥3 | 67 (45.6) | 51 (76.1) | 14 ((20.9) | 58 (86.6) |
| Number of days with prior antibiotics, *n/N’* (%) | *N*=147 | *N*=122 | *N*=27 | *N*=130 |
| ≤3 | 46 (31.3) | 41 (89.1) | 8 (17.4) | 41 (89.1) |
| 4–7 | 47 (32.0) | 39 (83.0) | 9 (19.1) | 42 (89.4) |
| >7 | 54 (36.7) | 42 (77.8) | 10 (18.5) | 47 (87.0) |
| Prior antibiotic treatment, *n/N’* (%) |  |  |  |  |
| Colistin | 81 (46.6) | 66 (81.5) | 17 (21.0) | 70 (86.4) |
| Meropenem | 55 (31.6) | 43 (78.2) | 12 (21.8) | 47 (85.5) |
| Ceftazidime-avibactam | 55 (31.6) | 43 (78.2) | 12 (21.8) | 46 (83.6) |
| Ceftolozane-tazobactam | 9 (5.2) | 7 (77.8) | 1 (11.1) | 8 (88.9) |
| Cefiderocol as first line, *n/N’* (%) |  |  |  |  |
| No | 149 (85.6) | 123 (82.6) | 28 (18.8) | 131 (87.9) |
| Yes | 25 (14.4) | 24 (96.0) | 2 (8.0) | 24 (96.0) |
| Combination treatment, *n/N’ (%)* |  |  |  |  |
| No | 118 (67.8) | 103 (87.3) | 18 (15.3) | 109 (92.4) |
| Yes | 56 (32.2) | 44 (78.6) | 12 (21.4) | 46 (82.1) |

*N’*, number of patients with available information in the category.
COVID-19, coronavirus disease-2019; EOT, end of treatment; ICU, intensive care unit; RRT, renal replacement therapy.
^a^Transplant recipient, immunosuppressive treatment (e.g. high-dose corticosteroids, calcineurin inhibitors, anti-CD20, IL-1 inhibitors and IL-6 inhibitors).
^b^Missing *N’*=23.
^c^Missing *N’*=10.

**Table S8.** Clinical cure, all-cause mortality at Day 28 and clinical success rates by pathogen and by infection site in the overall primary analysis population (*N*=261)

|  | Infection site | | | | | | | | |
| --- | --- | --- | --- | --- | --- | --- | --- | --- | --- |
|  | Overall | Respiratory | IAI | UTI | SSTI | BSI^a^ | Bone and joint | Other |  |
| Clinical cure at EOT, *n/N*’ (%) or *n/N’* (%) | |  |  |  |  |  |  |  |  |
| Overall | 210/261 (80.5) | 95/125 (76.0) | 26/38 (68.4) | 36/38 (94.7) | 24/26 (92.3) | 19/24 (79.2) | 6/6 (100) | 4/4 (100)^b^ |  |
| *P. aeruginosa* | 147/174 (84.5) | 64/81 (79.0) | 16/23 (69.6) | 27/28 (96.4) | 20/22 (90.9) | 11/11 (100) | 6/6 (100) | 3/3 (100)^c^ |  |
| *Pseudomonas* spp. | 12/15 (80.0) | 0/2 (0.0) | 2/3 (66.7) | 6/6 (100) | 3/3 (100) | 1/1 (100) | 0/0 (0) | 0/0 (0) |  |
| *K. pneumoniae* | 18/26 (69.2) | 12/16 (75.0) | 4/7 (57.1) | 0/0 (0) | 0/0 (0) | 2/3 (66.7) | 0/0 (0) | 0/0 (0) |  |
| Other Enterobacterales | 9/12 (75.0) | 5/5 (100) | 2/2 (100) | 0/1 (0) | 1/1 (100) | 1/3 (33.3) | 0/0 (0) | 0/0 (0) |  |
| *S. maltophilia* | 14/20 (70.0) | 7/11 (63.6) | 2/2 (100) | 1/1 (100) | 0/0 (0) | 3/5 (60.0) | 0/0 (0) | 1/1 (100)^d^ |  |
| *B. cepacia* complex | 5/8 (62.5) | 5/7 (71.4) | 0/1 (0) | 0/0 (0) | 0/0 (0) | 0/0 (0) | 0/0 (0) | 0/0 (0) |  |
| *Achromobacter* spp. | 4/5 (80) | 1/2 (50.0) | 0/0 (0) | 2/2 (100) | 0/0 (0) | 1/1 (100) | 0/0 (0) | 0/0 (0) |  |
| *R. mannitolilytica* | 1/1 (100) | 1/1 (100) | 0/0 (0) | 0/0 (0) | 0/0 (0) | 0/0 (0) | 0/0 (0) | 0/0 (0) |  |
| All-cause mortality at Day 28 by pathogen, *n/N* (%) or *n/N*’ (%) | | | |  |  |  |  |  |  |
| Overall | 56/261 (21.5) | 34/125 (27.2) | 9/38 (23.7) | 6/38 (15.8) | 2/26 (7.7) | 5/24 (20.8) | 0/6 (0) | 0/4 (0)^b^ |  |
| *P. aeruginosa* | 30/174 (17.2) | 18/81 (22.2) | 5/23 (21.7) | 4/28 (14.3) | 2/22 (9.1) | 1/11 (9.1) | 0/6 (0) | 0/3 (0)^c^ |  |
| *Pseudomonas* spp. | 2/15 (13.3) | 2/2 (100) | 0/3 (0) | 0/6 (0) | 0/3 (0) | 0/1 (0) | 0/0 (0) | 0/0 (0) |  |
| *K. pneumoniae* | 10//26 (38.5) | 6/16 (37.5) | 3/7 (42.9) | 0/0 (0) | 0/0 (0) | 1/3 (33.3) | 0/0 (0) | 0/0 (0) |  |
| Other Enterobacterales | 3/12 (25.0) | 1/5 (20.0) | 0/2 (0) | 1/1 (100) | 0/1 (0) | 1/3 (33.3) | 0/0 (0) | 0/0 (0) |  |
| *S. maltophilia* | 6/20 (30.0) | 4/11 (36.4) | 0/2 (0) | 0/1 (0) | 0/0 (0) | 2/5 (40.0) | 0/0 (0) | 0/1 (0)^d^ |  |
| *B. cepacia* complex | 3/8 (37.5) | 2/7 (28.6) | 1/1 (100) | 0/0 (0) | 0/0 (0) | 0/0 (0) | 0/0 (0) | 0/0 (0) |  |
| *Achromobacter* spp. | 2/5 (40.0) | 1/2 (50.0) | 0/0 (0) | 1/2(50) | 0/0 (0) | 0/1 (0) | 0/0 (0) | 0/0 (0) |  |
| *R. mannitolilytica* | 0/1 (0) | 0/1 (0) | 0/0 (0) | 0/0 (0) | 0/0 (0) | 0/0 (0) | 0/0 (0) | 0/0 (0) |  |
| Composite clinical success, *n/N* (%) or *n/N*’ (%) | | |  |  |  |  |  |  |  |
| Overall | 220/261 (84.3) | 100/125 (80.0) | 29/38 (76.3) | 36/38 (94.7) | 25/26 (96.2) | 20/24 (83.3) | 6/6 (100) | 4/4 (100)^b^ |  |
| *P. aeruginosa* | 155/174 (89.1) | 69/81 (85.2) | 18/23 (78.3) | 27/28 (96.4) | 21/22 (95.5) | 11/11 (100) | 6/6 (100) | 3/3 (100)^c^ |  |
| *Pseudomonas* spp. | 13/15 (86.7) | 0/2 (0.0) | 3/3 (100) | 6/6 (100) | 3/3 (100) | 1/1 (100) | 0/0 (0) | 0/0 (0) |  |
| *K. pneumoniae* | 18/26 (69.2) | 12/16 (75.0) | 4/7 (57.1) | 0/0 (0) | 0/0 (0) | 2/3 (66.7) | 0/0 (0) | 0/0 (0) |  |
| Other Enterobacterales | 10/12 (83.3) | 5/5 (100) | 2/2 (100) | 0/1 (0) | 1/1 (100) | 2/3 (66.7) | 0/0 (0) | 0/0 (0) |  |
| *S. maltophilia* | 14/20 (70.0) | 7/11 (63.6) | 2/2 (100) | 1/1 (100) | 0/0 (0) | 3/5 (60.0) | 0/0 (0) | 1/1 (100)^d^ |  |
| *B. cepacia* complex | 5/8 (62.5) | 5/7 (71.4) | 0/1 (0) | 0/0 (0) | 0/0 (0) | 0/0 (0) | 0/0 (0) | 0/0 (0) |  |
| *Achromobacter* spp. | 4/5 (80) | 1/2 (50.0) | 0/0 (0) | 2/2 (100) | 0/0 (0) | 1/1 (100) | 0/0 (0) | 0/0 (0) |  |
| *R. mannitolilytica* | 1/1 (100) | 1/1 (100) | 0/0 (0) | 0/0 (0) | 0/0 (0) | 0/0 (0) | 0/0 (0) | 0/0 (0) |  |

*N*, overall patient number; *N’*, total number of patients in the category.
BSI, bloodstream infection; EOT, end of treatment; IAI, intra-abdominal infection; SSTI, skin and soft tissue infection; UTI, urinary tract infection.
^a^Data are BSI of unknown origin/catheter-related BSI.
^b^Includes central nervous system infection (*n*=2) and mediastinitis (*n*=2).
^c^Includes central nervous system infection (*n*=2) and mediastinitis (*n*=1).
^d^Includes mediastinitis (*n*=1).

**Table S9.** Univariate and multivariate logistic regression analysis for clinical cure in patients with *P. aeruginosa* infections (*N*=174)

| Clinical cure | Univariate analysis (*N*=174) | | | Multivariate analysis (*N*=172) | | |
| --- | --- | --- | --- | --- | --- | --- |
|  | OR | 95% CI | Wald test *P* value | OR | 95% CI | Wald test *P* value |
| Age (continuous) | 0.98 | 0.94–1.01 | 0.1824 | 0.94 | 0.90–0.99 | 0.0144 |
| Sex (male vs female) | 0.85 | 0.27–2.27 | 0.7621 |  |  |  |
| CCI score (continuous) | 0.95 | 0.80–1.15 | 0.5830 |  |  |  |
| Septic shock (yes vs no) | 0.22 | 0.09–0.52 | 0.0006 | 0.22 | 0.08–0.58 | 0.0027 |
| Creatinine clearance (>30 mL/min vs ≤30 mL/min) | 0.84 | 0.23–2.42 | 0.7607 |  |  |  |
| RRT (yes vs no) | 0.29 | 0.12–0.70 | 0.0056 |  |  |  |
| Secondary bloodstream infection (yes vs no) | 0.39 | 0.15–1.07 | 0.0574 |  |  |  |
| Previously colonised (yes vs no) | 1.32 | 0.57–3.10 | 0.5145 |  |  |  |
| Infection (polymicrobial vs monomicrobial) | 0.56 | 0.23–1.47 | 0.2165 |  |  |  |
| Immunosuppression (yes vs no) | 0.86 | 0.35–2.35 | 0.7531 |  |  |  |
| COVID-19 (yes vs no) | 0.55 | 0.23–1.35 | 0.1782 |  |  |  |
| Prior antibiotics (yes vs no) | 0.2 | 0.01–1.0 | 0.1194 |  |  |  |
| Duration of prior GN antibiotic (continuous) | 0.96 | 0.91–1.01 | 0.1138 | 0.93 | 0.87–0.98 | 0.0092 |
| Duration of prior GN antibiotic (categorical)^a^ | 0.6 | 0.37–0.91 | 0.0223 |  |  |  |
| Mechanical ventilation at baseline (yes vs no) | 0.29 | 0.11–0.69 | 0.0064 | 0.35 | 0.11–0.98 | 0.0499 |
| Mortality (>20% vs <20%) | 0.2 | 0.06–0.71 | 0.0121 |  |  |  |

CCI, Charlson Comorbidity Index; CI, confidence interval; COVID-19, coronavirus disease-2019; GN, Gram-negative; OR, odds ratio; RRT, renal replacement therapy.
^a^1: 0 days; 2: 1–3 days; 3: 4–7 days; 4: >7 days.
